# Red Flag Signs and Symptoms for Patients With Early-Onset Colorectal Cancer: A Systematic Review and Meta-Analysis

**DOI:** 10.1001/jamanetworkopen.2024.13157

**Published:** 2024-05-24

**Authors:** Joshua Demb, Jennifer M. Kolb, Jonathan Dounel, Cassandra D. L. Fritz, Shailesh M. Advani, Yin Cao, Penny Coppernoll-Blach, Andrea J. Dwyer, Jose Perea, Karen M. Heskett, Andreana N. Holowatyj, Christopher H. Lieu, Siddharth Singh, Manon C. W. Spaander, Fanny E. R. Vuik, Samir Gupta

**Affiliations:** 1 Division of Gastroenterology, Department of Medicine, University of California, San Diego, La Jolla; 2 Vatche and Tamar Manoukian Division of Digestive Diseases, David Geffen School of Medicine at UCLA, VA Greater Los Angeles Healthcare System, Los Angeles, California; 3 Department of Medicine, University of California San Diego, La Jolla; 4 Division of Gastroenterology, Washington University in St Louis, St Louis, Missouri; 5 Department of Internal Medicine, Roswell Park Comprehensive Cancer Center, Buffalo, New York; 6 Division of Public Health Sciences, Department of Surgery, Washington University School of Medicine, St Louis, Missouri; 7 UC San Diego Library, University of California San Diego, La Jolla; 8 University of Colorado Cancer Center, Colorado School of Public Health, Aurora; 9 Molecular Medicine Unit, Department of Medicine, Biomedical Research Institute of Salamanca, University of Salamanca, Salamanca, Spain; 10 Vanderbilt-Ingram Cancer Center, Vanderbilt University Medical Center, Vanderbilt University School of Medicine, Nashville, Tennessee; 11 Division of Medical Oncology, University of Colorado Denver Anschutz Medical Campus, Aurora; 12 Division of Biomedical Informatics, Department of Medicine, University of California San Diego, La Jolla; 13 Department of Gastroenterology and Hepatology, Erasmus University Medical Center, Rotterdam, the Netherlands; 14 Jennifer Moreno Veteran Affairs San Diego Healthcare System, San Diego, California; 15 Division of Gastroenterology, Department of Medicine, Washington University School of Medicine, St Louis, Missouri; 16 Alvin J. Siteman Cancer Center, Washington University School of Medicine, St Louis, Missouri; 17 Surgery Department, Vithas Arturo Soria University Hospital, Madrid, Spain

## Abstract

**Importance:**

Early-onset colorectal cancer (EOCRC), defined as a diagnosis at younger than age 50 years, is increasing, and so-called red flag signs and symptoms among these individuals are often missed, leading to diagnostic delays. Improved recognition of presenting signs and symptoms associated with EOCRC could facilitate more timely diagnosis and impact clinical outcomes.

**Objective:**

To report the frequency of presenting red flag signs and symptoms among individuals with EOCRC, to examine their association with EOCRC risk, and to measure variation in time to diagnosis from sign or symptom presentation.

**Data Sources:**

PubMed/MEDLINE, Embase, CINAHL, and Web of Science were searched from database inception through May 2023.

**Study Selection:**

Studies that reported on sign and symptom presentation or time from sign and symptom presentation to diagnosis for patients younger than age 50 years diagnosed with nonhereditary CRC were included.

**Data Extraction and Synthesis:**

Data extraction and quality assessment were performed independently in duplicate for all included studies using Preferred Reporting Items for Systematic Reviews and Meta-analyses reporting guidelines. Joanna Briggs Institute Critical Appraisal tools were used to measure risk of bias. Data on frequency of signs and symptoms were pooled using a random-effects model.

**Main Outcomes and Measures:**

Outcomes of interest were pooled proportions of signs and symptoms in patients with EOCRC, estimates for association of signs and symptoms with EOCRC risk, and time from sign or symptom presentation to EOCRC diagnosis.

**Results:**

Of the 12 859 unique articles initially retrieved, 81 studies with 24 908 126 patients younger than 50 years were included. The most common presenting signs and symptoms, reported by 78 included studies, were hematochezia (pooled prevalence, 45% [95% CI, 40%-50%]), abdominal pain (pooled prevalence, 40% [95% CI, 35%-45%]), and altered bowel habits (pooled prevalence, 27% [95% CI, 22%-33%]). Hematochezia (estimate range, 5.2-54.0), abdominal pain (estimate range, 1.3-6.0), and anemia (estimate range, 2.1-10.8) were associated with higher EOCRC likelihood. Time from signs and symptoms presentation to EOCRC diagnosis was a mean (range) of 6.4 (1.8-13.7) months (23 studies) and a median (range) of 4 (2.0-8.7) months (16 studies).

**Conclusions and Relevance:**

In this systematic review and meta-analysis of patients with EOCRC, nearly half of individuals presented with hematochezia and abdominal pain and one-quarter with altered bowel habits. Hematochezia was associated with at least 5-fold increased EOCRC risk. Delays in diagnosis of 4 to 6 months were common. These findings highlight the need to identify concerning EOCRC signs and symptoms and complete timely diagnostic workup, particularly for individuals without an alternative diagnosis or sign or symptom resolution.

## Key Points

**Question:**

In patients with early-onset colorectal cancer (EOCRC), what are the most common presenting signs and symptoms, what is their association with EOCRC risk, and what is the time from presentation to diagnosis?

**Findings:**

In this systematic review and meta-analysis including 81 studies and more than 24.9 million patients, nearly half of individuals with EOCRC presented with hematochezia and abdominal pain and one-quarter presented with altered bowel habits. Delays in diagnosis of 4 to 6 months from time of initial presentation were common.

**Meaning:**

These findings underscore the need to identify signs and symptoms concerning for EOCRC and complete timely diagnostic workup for individuals without an alternative diagnosis or sign or symptom resolution.

## Teaser

This systematic review and meta-analysis examines the frequency of presenting red flag signs and symptoms among individuals with early-onset colorectal (EOCRC), the associations of these signs and symptoms with risk of EOCRC, and variations in time from sign or symptom presentation to diagnosis.

## Introduction

The incidence of early-onset colorectal cancer (EOCRC), defined as a diagnosis at younger than age 50 years, has been increasing at an alarming rate, in contrast to the decreasing CRC rate among older individuals.^1^ These trends have been observed globally,^2,3,4,5,6,7,8,9^ and EOCRC rates in the US are projected to increase by at least 140% by 2030.^10^ These worrisome epidemiologic findings prompted an update in US CRC screening guidelines to begin screening among individuals at average risk at age 45 years.^11^

Outside of screening, early detection of symptomatic EOCRC remains a priority. Delayed diagnosis may be a result of late patient presentation and lack of clinician knowledge of common CRC symptoms, such as hematochezia or abdominal and pelvic pain, and signs, such as iron deficiency anemia. Patients and clinicians alike may downplay symptom severity and fail to recognize key red flags and clinical cues that should trigger suspicion of CRC.^12,13,14,15^ Furthermore, diagnostic algorithms in adults younger than 50 years often favor a less invasive and more conservative watchful waiting strategy, which could result in missed opportunities for intervention.^16^ Therefore, defining the prevalence of these common signs and symptoms and their associated EOCRC risk is a critical first step to inform care pathways.

Additionally, delays in diagnostic workup after sign or symptom presentation are up to 40% longer in younger compared with older individuals with CRC, which may contribute to greater proportion of late stage diagnosis (58%-89% vs 30%-63%) and increasing EOCRC mortality rates in the US (1.3% per year from 2008-2017).^17,18,19,20^ Mitigation strategies to expedite timely diagnoses may help decrease EOCRC morbidity and mortality. To address these gaps and pressing clinical issues, we performed a systematic review and meta-analysis to quantify the prevalence of signs and symptoms at EOCRC presentation, their association with EOCRC risk, and time to diagnosis.

## Methods

We conducted a systematic review and meta-analysis to answer 3 questions. First, which signs and symptoms are most commonly present in individuals diagnosed with EOCRC? Second, what is the association between EOCRC sign or symptom exposure and EOCRC risk? Third, what is the time from sign or symptom presentation to diagnosis of EOCRC? This study is registered on Prospero (identifier: CRD42020181296). We followed the Preferred Reporting Items for Systematic Reviews and Meta-analyses (PRISMA) reporting guideline.

### Data Sources and Search Strategy

A comprehensive literature search was performed in PubMed/MEDLINE, Embase, CINAHL, and Web of Science Core Collection from inception through May 2023 to identify candidate studies for inclusion (eTable 1 in Supplement 1). Results were exported and deduplicated in EndNote (Clarivate) using the Bramer method.^21^

### Study Selection and Inclusion and Exclusion Criteria

Study review and data extraction were performed in Covidence (Veritas Health Innovation). Two independent reviewers (among J. Demb, J.M.K, J. Dounel, C.D.L.F., S.M.A. and F.E.R.V.) screened titles and abstracts for eligibility and reviewed the full text of all designated articles, with a third reviewer (S.G.) providing consensus if needed. Studies that reported on sign or symptom presentation or time to diagnosis for patients younger than age 50 years diagnosed with nonhereditary CRC were included. Studies with fewer than 15 eligible patients, most patients younger than age 18 years, or published before 1996 or in which more than half of the study period occurred before 1996—the year when EOCRC incidence rates began increasing, notably among adults aged 40 to 49 years—were excluded.^22^ Meeting abstracts, reviews, non-English articles, and nonoriginal research were excluded.

### Data Extraction and Risk of Bias Assessment

Two reviewers (J. Demb and J.M.K.) extracted relevant data from articles meeting inclusion criteria, including study characteristics (time period, design, country, and population composition), the proportion of patients with EOCRC presenting with each sign and symptom, relative estimates for association of signs and symptoms with EOCRC risk, and time from symptom presentation to diagnosis, as defined by either patient report of onset of symptoms or medical record capture of symptom presentation. Risk of bias assessment was performed using the Joanna Briggs Institute (JBI) Critical Appraisal tools for cohort studies, cross-sectional studies, and case-control studies.^23^ These tools include questions characterizing a study’s sources of bias and internal validity, measurement of exposures, outcomes and follow-up, and potential risk of selection or information bias. Risk of bias was graded and separated into 3 categories: low risk, 75% to 100% of checklist items included; moderate risk, 50% to 75% of checklist items; and high risk, less than 50% of checklist items.

### Statistical Analysis

For the assessment of signs and symptoms among patients with EOCRC, sign and symptom proportions were pooled individually across studies and proportions were compared using forest plots. Pooled prevalence estimates were calculated via random-effects meta-analysis using the Hartung and Knapp method, which has been found to perform well when between-study heterogeneity is high and study sample sizes are similar.^24,25^ Stratified analyses were performed to measure pooled estimates based on specific study characteristics to assess potential variations in estimates, including geographic study location (US vs non-US), study age groups (≤40 years and ≤50 years), risk of bias (low, moderate, high), and data source type (claims or medical record, patient-reported, not well defined). Meta-regression was also performed adjusting for percentage of male study participants and the year of study publication.

We assessed heterogeneity between study-specific estimates using the inconsistency index (*I*^2^), and used cutoffs of 0% to 30%, 30% to 60%, 60% to 90%, and 90% to 100% to suggest minimal, moderate, substantial, and considerable heterogeneity, respectively. Between-study sources of heterogeneity were investigated using subgroup analyses by stratifying original estimates according to study characteristics. In this analysis, *P* < .10 differences between subgroups was considered statistically significant (ie, a value of *P* < .10 suggests that stratifying based on that particular study characteristic partly explained the heterogeneity observed in the analysis).

Signs and symptoms with estimates of EOCRC risk across at least 3 studies were described using forest plots. Due to significant heterogeneity across studies, particularly the composition of the analytic samples, we were unable to conduct meta-analysis of signs and symptoms and their association with EOCRC risk. Time to diagnosis was defined as the date of sign or symptom presentation to the date of diagnosis and stratified according to the data source type, since this was measured differently across studies. These data were aggregated based on whether the estimate was a mean or median, and the distributions of mean and median times to diagnosis were evaluated.

*P* values were 2-sided, and statistical significance was set at *P* < .10. All analyses were performed using R statistical software version 4.1.3 (R Project for Statistical Computing), with plots and statistical analyses calculated using the suite of functions and commands within the meta package and the ggplot2 package, with R code provided in the eMethods in Supplement 1.^26^ Data were analyzed from August 2022 and April 2024.

## Results

### Search Strategy and Study Characteristics

**Figure 1.**
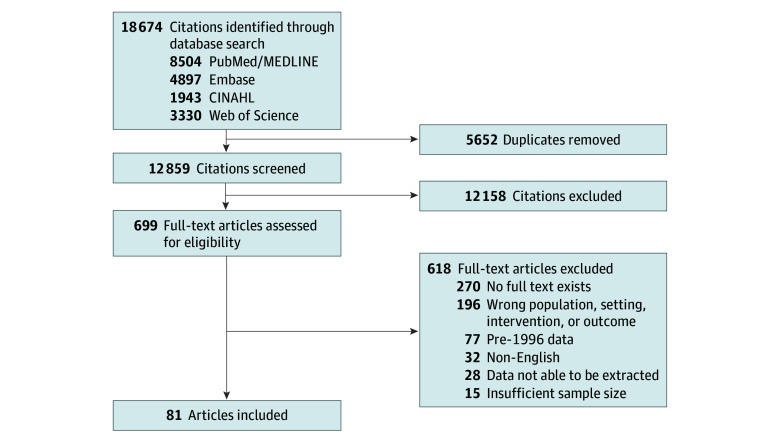
Flowchart of Study Inclusion

Of the 12 859 unique articles retrieved, 699 full texts were reviewed, and 81 studies^12,13,18,27,28,29,30,31,32,33,34,35,36,37,38,39,40,41,42,43,44,45,46,47,48,49,50,51,52,53,54,55,56,57,58,59,60,61,62,63,64,65,66,67,68,69,70,71,72,73,74,75,76,77,78,79,80,81,82,83,84,85,86,87,88,89,90,91,92,93,94,95,96,97,98,99,100,101,102,103,104^ were included (Figure 1 and Table). There were 76 cross-sectional studies,^12,13,18,27,28,29,30,31,32,33,34,35,37,38,39,40,41,42,43,45,46,48,49,50,51,52,53,54,55,56,57,58,59,60,61,62,63,64,65,66,67,68,69,70,71,72,73,74,75,76,77,78,79,80,81,82,83,84,85,86,87,88,89,90,91,92,94,96,97,98,99,100,101,102,103,104^ 4 case-control studies,^44,47,93,95^ and 1 cohort study.^36^ Studies were performed in Africa (5 studies),^31,41,54,65,84^ Asia or the Middle East (26 studies),^18,35,37,42,48,49,51,52,53,56,62,66,67,71,77,78,80,82,85,96,99,100,101,102,103,104^ Europe (19 studies),^28,29,40,43,45,46,50,55,57,58,60,63,64,72,73,75,87,91,93^ North America (23 studies),^12,27,32,33,34,36,39,44,47,59,61,68,69,70,74,81,86,88,92,94,95,97,98^ South America (5 studies),^30,38,83,89,90^ and Oceania (2 studies).^76,79^ There were 67 studies^12,13,27,28,30,32,33,34,36,38,39,40,44,45,47,48,51,52,53,54,55,56,57,58,59,60,61,62,63,64,65,66,67,68,69,70,71,72,73,74,75,76,77,79,81,83,84,86,87,88,89,90,91,92,93,94,95,96,97,98,99,100,101,102,104^ deemed to have low risk of bias, 10 studies^18,29,31,35,37,46,50,80,85,103^ with moderate risk of bias, and 4 studies^41,49,78,82^ with high risk of bias, based on JBI checklists. Notable sources of bias included using patient-reported or inadequately defined measures of signs or symptoms and time to diagnosis (eTable 2 in Supplement 1).

**Table.** Publication and Outcomes Data for All Included Studies

Source	Country	Study period	Study design	Patients aged <50 y, No.	Study population	Risk of bias	Outcome addressed (data source)
Al-Barrak et al,^27^ 2011	Canada	January 1985 to December 2005	Cross-sectional	62	Patients with CRC aged ≤30 y referred to British Columbia Cancer Agency	Low	Symptoms at presentation (claims and medical records)
Arhi et al,^28^ 2019	UK	2006-2013	Cross-sectional	508	Patients aged <50 y with CRC diagnosis (*ICD-O-3* 18-20) in Clinical Practice Research Datalink cancer registry	Low	Symptoms at presentation (claims and medical records) Time to diagnosis (claims and medical records)
Arriba et al,^29^ 2019	Spain	NR	Cross-sectional	98	Patients diagnosed at age ≤50 y at Hospital Universitario 12 de Octubre in Madrid	Moderate	Symptoms at presentation (patient reported) Time to diagnosis (patient reported)
Avellaneda et al,^30^ 2021	Argentina	January 2015 to May 2020	Cross-sectional	32	Patients aged <50 y at an academic hospital in Buenos Aires, Argentina, using the surgery department’s database	Low	Symptoms at presentation (claims and medical records)
Ben-Ishay et al,^18^ 2013	Israel	January 2000 to December 2009	Cross-sectional	31	Patients under the aged <50 y admitted to Department of General Surgery at Rambam Health Care Campus, Haifa	Moderate	Symptoms at presentation (patient reported) Time to diagnosis (patient reported)
Bouassida et al,^31^ 2012	Tunisia	2001-2010	Cross-sectional	40	Records of 280 patients aged <40 y and ≥41 y with CRC who were referred between 2001 and 2010 to the Department of Surgery, Hospital of Nabeul	Moderate	Symptoms at presentation (not well defined) Time to diagnosis (not well defined)
Castelo et al,^32^ 2023	Canada	October 2003 to December 2018	Cross-sectional	6853	Ontario residents aged 15-49 y using the Ontario Cancer Registry	Low	Symptoms at presentation (claims and medical records) Time to diagnosis (claims/medical records)
Cercek et al,^12^ 2021	US	January 2014 to June 2019	Cross-sectional	759	Patients aged <35 y and 36-49 y at Memorial Sloan Kettering Cancer Center	Low	Symptoms at presentation (claims and medical records)
Chen et al,^33^ 2017	US	January 2008 to December 2014	Cross-sectional	253	Patients aged <50 y with colorectal adenocarcinoma at Stanford Cancer Institute	Low	Symptoms at presentation (claims and medical records) Time to diagnosis (patient reported and medical records)
Chiu et al,^34^ 2023	US	January 2000 to May 2020	Cross-sectional	103	Non-Hispanic Black and Non-Hispanic White patients aged <50 y with primary CRC who received care at Boston Medical Center	Low	Symptoms at presentation (claims and medical records) Time to diagnosis (patient reported and medical records)
Chou et al,^35^ 2011	Taiwan	2001-2006	Cross-sectional	69	Patients aged 22-40 y with CRC at Taipei Veterans General Hospital	Moderate	Symptoms at presentation (not well defined)
Demb et al,^36^ 2021	US	1996-2016	Cohort	892 740 (239 000 IDA and 653 740 hematochezia)	US veterans aged 18-49 y receiving VHA care	Low	Strength of association (claims and medical records)
De Silva et al,^37^ 2000	Sri Lanka	1982-1997	Cross-sectional	60	Patients aged 18-40 y with confirmed CRC in records of University Department of Pathology, Colombo, where all patients had undergone surgery at the University Surgical Unit, National Hospital, Sri Lanka	Moderate	Symptoms at presentation (patient reported) Time to diagnosis (patient reported)
De Sousa et al,^38^ 2014	Brazil	January 2006 to December 2010	Cross-sectional	66	Patients aged <50 y with a histopathological diagnosis of adenocarcinoma with primary tumor site at the colon or rectum in whom colonoscopy was indicated because of clinical symptoms and who were treated at 1 institution in Brazil	Low	Symptoms at presentation (claims and medical records) Time to diagnosis (patient reported and medical records)
Dharwadkar et al,^39^ 2021	US	January 2009 to June 2017	Cross-sectional	319	Patients aged <50 y diagnosed with or treated for histologically confirmed CRC at a large, integrated safety-net health system in Dallas, Texas	Low	Symptoms at presentation (claims and medical records)
Di Leo et al,^40^ 2021	Italy	January 2015 to December 2018	Cross-sectional	54	Individuals aged 18-49 y consulted for CRC in a tertiary academic medical center in Milan	Low	Symptoms at presentation (claims and medical records) Time to diagnosis (claims/medical records)
El-Hennawy et al,^41^ 2003	Egypt	June 1998 to June 2001	Cross-sectional	26	Patients aged <40 y treated at Alexandria Main University Hospital in Alexandria, Egypt	High	Symptoms at presentation (not well defined) Time to diagnosis (not well defined)
Fayaz et al,^42^ 2018	Kuwait	January 2000 to December 2007	Cross-sectional	130	Patients aged ≤50 y identified in the medical index of the Kuwait Cancer Control Center for colonic adenocarcinoma	Low	Symptoms at presentation (claims and medical records)
Foppa et al,^43^ 2021	Italy	January 2008 to October 2019	Cross-sectional	101	Data from patients aged 18-39 y who underwent surgery were retrospectively collected from prospectively maintained databases of 3 European tertiary centers	Low	Symptoms at presentation (claims/medical records) Time to diagnosis (claims/medical records)
Fritz et al,^44^ 2023	US	2006-2015	Case-control	5075	Patients aged 18-49 y identified using the IBM MarketScan Commercial Database	Low	Symptoms at presentation (claims and medical records) Strength of association (claims and medical records) Time to diagnosis (claims and medical records)
Frostberg et al,^45^ 2020	Denmark	2001-2013	Cross-sectional	521 (174 between 2010-2013)	Patients with early-onset colorectal cancer were defined as patients diagnosed with histologically verified colon or rectal cancer at aged 18-40 y; patients were identified in the Danish Colorectal Cancer Group and Danish Cancer Registry	Low	Symptoms at presentation (claims and medical records)
Ganapathi et al,^46^ 2011	UK	January 1990 to December 2009	Cross-sectional	59	Patients aged ≤40 y with histological diagnosis of CRC at St George’s Hospital, London	Moderate	Symptoms at presentation (claims and medical records or patient-reported)
Glover et al,^47^ 2019	US	July 2013 to July 2018	Case-control	1680	Patients aged 20-39 y with first diagnosis of CRC between 2013 and 2018 based on the Systematized Nomenclature of Medicine-Clinical Terms identified from a commercial database (Explorys)	Low	Symptoms at presentation (claims and medical records) Strength of association (claims and medical records)
Goh et al,^48^ 2020	Singapore	2010-2017	Cross-sectional	99	Patients aged 18-49 y at a tertiary hospital in Singapore	Low	Symptoms at presentation (claims and medical records)
Gul et al,^49^ 2012	Pakistan	January 2007 to June 2007	Cross-sectional	50	Patients aged <40 y selected from Surgical Department, Khyber Teaching Hospital in Peshawar	High	Symptoms at presentation (not well defined)
Gunel et al,^50^ 2001	Turkey	1993-1998	Cross-sectional	100	Patients aged ≤50 y admitted to an oncology center in Turkey	Moderate	Symptoms at presentation (not well defined) Time to diagnosis (not well defined)
Haleshappa et al,^51^ 2017	India	2010-2014	Cross-sectional	89	Patients aged <40 y in tumor registry at a hospital in India	Low	Symptoms at presentation (claims and medical records)
Haresh et al,^52^ 2016	India	2007-2013	Cross-sectional	60	Patients aged 15-34 y with rectal cancer at the All India Institute	Low	Symptoms at presentation (claims and medical records) Time to diagnosis (claims and medical records)
Haroon et al,^53^ 2013	Pakistan	1994-2004	Cross-sectional	23	Patients aged 15-40 y presenting with histopathological diagnosis of carcinoma rectum at the Aga Khan University Hospital	Low	Symptoms at presentation (claims and medical records) Time to diagnosis (claims and medical records)
Jarrar et al,^54^ 2022	Tunisia	January 2002 to December 2014	Cross-sectional	67	Patients aged <50 y in the Department of General and Digestive Surgery in Farhat Hached University Hospital of Sousse	Low	Symptoms at presentation (claims and medical records) Time to diagnosis (claims and medical records)
Josifovski et al,^55^ 2004	Serbia	January 1998 to December 2002	Cross-sectional	19	Patients aged 25-40 y with sporadic colon cancer treated at the Institute of Oncology and Radiology of Serbia, Beograd	Low	Symptoms at presentation (claims and medical records)
Kansakar et al,^56^ 2012	Nepal	January 1999 to December 2008	Cross-sectional	62	Patients aged 20-39 y with CRC at Tribhuvan University Teaching Hospital, Kathmandu, Nepal	Low	Symptoms at presentation (claims and medical records) Time to diagnosis (claims and medical records)
Kaplan et al,^57^ 2013	Turkey	May 2003 to June 2010	Cross-sectional	56	Patients aged 20-25 y diagnosed with CRC at referral medical oncology centers in Turkey	Low	Symptoms at presentation (claims and medical records) Time to diagnosis (claims and medical records)
Kaplan et al,^58^ 2019	Turkey	May 2003 to December 2015	Cross-sectional	141	Patients aged 20-25 y diagnosed with CRC at referral centers in Turkey	Low	Symptoms at presentation (claims and medical records) Time to diagnosis (claims and medical records)
Karsten et al,^59^ 2008	US	January 1998 to December 2005	Cross-sectional	41	Patients aged 19-40 y from tumor registry at Harbor-UCLA Medical Center	Low	Symptoms at presentation (claims and medical records)
Kocian et al,^60^ 2017	Czech Republic	2005-2015	Cross-sectional	38	Patients aged <40 y with CRC treated at the Department of Surgery at Motol University Hospital in Prague	Low	Symptoms at presentation (claims and medical records)
Lapumnuaypol et al,^61^ 2018	US	January 1997 to December 2016	Cross-sectional	109	Patients aged 20-49 y diagnosed with CRC and admitted at Einstein Medical Center, Philadelphia, Pennsylvania	Low	Symptoms at presentation (claims and medical records) Time to diagnosis (claims and medical records)
Law et al,^62^ 2017	Singapore	January 2007 to December 2015	Cross-sectional	154	Patients aged 19-49 y diagnosed with CRC at a single institution	Low	Symptoms at presentation (claims and medical records) Time to diagnosis (claims and medical records)
Leff et al,^63^ 2007	UK	1982-1992	Cross-sectional	49	Patients aged ≤40 y diagnosed with CRC at St Mark’s Hospital; 67% of patients were aged 31-40 y, and 2 patients presented with CRC in their teens	Low	Symptoms at presentation (claims and medical records)
Leopa et al,^64^ 2023	Romania	January to December 2018	Cross-sectional	81	Patients aged <40 y who had undergone surgery for colon cancer in the General Surgery Clinic of the Constanta County Emergency Clinical Hospital.	Low	Symptoms at presentation (claims and medical records) Time to diagnosis (claims and medical records)
Limaiem et al,^65^ 2018	Tunisia	April 2000 to November 2014	Cross-sectional	32	Patients aged <40 y diagnosed at the pathology department of Mongi Slim Hospital	Low	Symptoms at presentation (claims and medical records)
Lin et al,^66^ 2005	Taiwan	1992-2002	Cross-sectional	45	Patients aged 18-39 y treated at Taipei Veterans General Hospital	Low	Symptoms at presentation (claims and medical records)
Makmun et al,^67^ 2021	Indonesia	January 2008 to December 2019	Cross-sectional	205	Patients aged 18-49 y at a tertiary academic hospital in Jakarta	Low	Symptoms at presentation (claims and medical records)
Melendez-Rosado et al,^68^ 2022	US	2010-2016	Cross-sectional	56	Patients aged ≤40 y diagnosed with colorectal malignant neoplasms during 2010-2016 at a single institution	Low	Symptoms at presentation (claims and medical records)
Mogor et al,^69^ 2019	US	2010-2012	Cross-sectional	2748	Patients aged <50 y with rectal cancer identified from the NIS database using *ICD-9-CM* code 48	Low	Symptoms at presentation (claims and medical records)
Myers et al,^70^ 2013	US	July 1996 to May 2012	Cross-sectional	180	Patients aged 17-49 y who underwent CRC operations at 2 institutions in New York, New York	Low	Symptoms at presentation (claims and medical records)
Nagai et al,^71^ 2016	Japan	January 2005 to December 2011	Cross-sectional	70	Patients aged 30-49 y with CRC who underwent surgical resection at University of Tokyo	Low	Symptoms at presentation (claims and medical records) Time to diagnosis (claims and medical records)
Nikolic et al,^72^ 2023	Serbia	January 2009 to December 2019	Cross-sectional	87	Patients aged 18-39 y at the Institute for Oncology and Radiology of Serbia	Low	Symptoms at presentation (claims and medical records)
Ozaydin et al,^73^ 2019	Turkey	2000-2017	Cross-sectional	32	Patients aged ≤30 y, with 50% aged <18 y	Low	Symptoms at presentation (claims and medical records)
Park et al,^74^ 2022	US	January 2004 to June 2019	Cross-sectional	3856	Patients aged 20-49 y evaluated at Memorial Sloan Kettering Cancer Center, identified by *ICD-O-3* site and histology codes	Low	Symptoms at presentation (claims and medical records)
Patel et al,^75^ 2016	UK	December 2008 to May 2014	Cross-sectional	18	Patients aged 37-49 y referred by general practitioners for suspected CRC at West Suffolk Hospital and later confirmed with CRC	Low	Symptoms at presentation (claims and medical records) Time to diagnosis (claims and medical records)
Plunkett et al,^76^ 2014	New Zealand	January 1997 to December 2007	Cross-sectional	50	Patients aged ≤25 y with CRC from the New Zealand Cancer Registry	Low	Symptoms at presentation (claims and medical records)
Poudyal et al,^77^ 2017	Nepal	July 2015 to April 2017	Cross-sectional	30	Patients aged ≤40 y with colonoscopically diagnosed and histopathologically proven cases of colon cancer in Bir Hospital	Low	Symptoms at presentation (claims and medical records)
Quach et al,^78^ 2012	Vietnam	March 2009 to March 2011	Cross-sectional	112	Patients aged 17-49 y who underwent colonoscopy, University Medical Center Ho Chi Minh	High	Symptoms at presentation (not well defined) Time to diagnosis (not well defined)
Rajagopalan et al,^79^ 2021	Australia	2011-2019	Cross-sectional	75	Patients aged 18-45 y who had surgical resection at a surgery unit in Dandenon Hospital, Victoria	Low	Symptoms at presentation (claims and medical records)
Raman et al,^80^ 2014	India	2003-2011	Cross-sectional	72	Patients aged ≤50 y with CRC undergoing surgical resection in 4 tertiary cancer care hospitals in Hyderabad, India	Moderate	Symptoms at presentation (patient reported) Time to diagnosis (patient reported)
Reddy et al,^81^ 2021	US	August 2008 to December 2016	Cross-sectional	139	Patients aged 18-49 y at Carilion Roanoke Memorial Hospital	Low	Symptoms at presentation (claims and medical records) Time to diagnosis (claims/medical records)
Rho et al,^13^ 2017	International	June 2003 to June 2014	Cross-sectional	224	Patients aged 24-44 y with pathologically proven adenocarcinoma of the colon or rectum included from 6 international tertiary cancer centers (Canada, Italy, Czech Republic, Ireland, and Bulgaria)	Low	Symptoms at presentation (claims and medical records)
Riaz et al,^82^ 2017	Pakistan	August 2014 to January 2016	Cross-sectional	105	Patients aged 18-49 y with histology records and interview data from different government hospitals in Islamabad and Rawalpindi	High	Symptoms at presentation (patient reported)
Ruiz et al,^83^ 2016	Peru	January 2005 to December 2010	Cross-sectional	196	Patients aged ≤40 y with CRC diagnosed at Instituto Nacional de Enfermedades Neoplásicas	Low	Symptoms at presentation (claims and medical records) Time to diagnosis (claims and medical records)
Saidi et al,^84^ 2018	Kenya	1993-2005	Cross-sectional	70	Patients aged ≤40 y with CRC at Kenyatta National Hospital in Nairobi, Kenya; patient age range, 10-40 y (mean [SD], 30.1 [6.9] y)	Low	Symptoms at presentation (claims and medical records) Time to diagnosis (claims and medical records)
Saluja et al,^85^ 2014	India	2003-2013	Cross-sectional	66	Patients aged 20-40 y who attended the outpatient department of a surgical unit and received treatment in the form of surgery, preoperative neoadjuvant therapy, adjuvant therapy, or palliative chemotherapy	Moderate	Symptoms at presentation (not well defined) Time to diagnosis (not well defined)
Sandhu et al,^86^ 2020	US	2012-2018	Cross-sectional	173	Patients aged <50 y at University of Colorado in the cancer center registry	Low	Symptoms at presentation (claims and medical records) Time to diagnosis (claims and medical records)
Schellerer et al,^87^ 2012	Germany	January 1996 to December 2005	Cross-sectional	244	Patients aged ≤50 y (range, 12-50 y) who received tumor resection for CRC at a single institution	Low	Symptoms at presentation (claims and medical records)
Scott et al,^88^ 2016	US	1997-2007	Cross-sectional	56	Patients aged ≤50 y treated for rectal cancer at University of Vermont Medical Center identified from American College of Surgeons Commission on Cancer certified tumor registry	Low	Symptoms at presentation (claims and medical records) Time to diagnosis (claims and medical records)
Silva et al,^89^ 2019	Brazil	January 2011 to November 2016	Cross-sectional	781	Patients aged 17-49 y with CRC at Institutodo Câncerdo Estadode São Paulo, Universidade de São Paulo	Low	Symptoms at presentation (claims and medical records)
Silva et al,^90^ 2020	Brazil	January 2013 to January 2018	Cross-sectional	39	Patients aged 20-49 y treated at Asa Norte Regional Hospital	Low	Symptoms at presentation (claims and medical records) Time to diagnosis (claims and medical records)
Singh et al,^91^ 2020	UK	January 2005 to December 2013	Cross-sectional	22	Patients aged <50 y with emergency presentation to West Suffolk Hospital	Low	Symptoms at presentation (claims and medical records)
Skalitsky et al,^92^ 2023	US	2005-2019	Cross-sectional	286	Patients aged <50 y identified via a retrospectively maintained database at the University of Iowa, a national cancer institute	Low	Symptoms at presentation (claims and medical records) Time to diagnosis (claims and medical records)
Stapley et al,^93^ 2017	UK	January 2000 to December 2013	Case-control	1680	Patients aged 18-49 y identified from data collected prospectively from the Clinical Practice Research Datalink	Low	Strength of association (claims and medical records)
Strum et al,^94^ 2019	US	January 2006 to May 2017	Cross-sectional	109	Patients aged 18-49 y from Scripps Green Hospital, La Jolla, California	Low	Symptoms at presentation (claims and medical records) Time to diagnosis (claims and medical records)
Syed et al,^95^ 2019	US	January 2012 to December 2016	Case-control	5710	Patients aged 25-49 y identified using the national database Explorys	Low	Symptoms at presentation (claims and medical records) Strength of association (claims and medical records)
Trivedi et al,^96^ 2022	India	January 2017 to December 2019	Cross-sectional	148	Patients aged <50 y at a tertiary cancer hospital in Patna, India	Low	Symptoms at presentation (claims and medical records) Time to diagnosis (claims and medical records)
Vajrevelu et al,^97^ 2021	US	2004-2018	Cross-sectional	6163	Patients aged 18-49 y from Clinformatics Data Mart Database (Optum)	Low	Symptoms at presentation (claims and medical records)
Vakil et al,^98^ 2021	US	1985-2017	Cross-sectional	637	Patients aged 18-44 y and 45-49 y with confirmed CRC from a cancer database of a large integrated health care system composed of 15 hospitals, 20 outpatient oncology clinics	Low	Symptoms at presentation (claims and medical records)
Wan lbrahim et al,^99^ 2020	Malaysia	January 2007 to December 2017	Cross-sectional	893	Patients aged <50 y from all 18 public and private hospitals in 3 states in northern Malaysia (Perlis, Kedah, and Penang)	Low	Symptoms at presentation (claims and medical records)
Wong et al,^100^ 2021	Malaysia	2002-2016	Cross-sectional	178	Patients aged <50 y diagnosed at University of Malaya Medical Center in Malaysia	Low	Symptoms at presentation (claims and medical records)
Zahir et al,^101^ 2014	Pakistan	January 2004 to December 2011	Cross-sectional	131	Patients aged 16-45 y with newly diagnosed CRC who presented to the Oncology Department, Aga Khan University Hospital, Karachi	Low	Symptoms at presentation (claims and medical records)
Zhang et al,^102^ 2009	China	January 1987 to December 2006 (2 groups: 1987-1996 and 1997-2006)	Cross-sectional	488	Patients aged 0-44 y who received colonoscopy in the Endoscopy Unit of The First Affiliated Hospital, Sun Yat-sen University	Low	Symptoms at presentation (claims and medical records)
Zhao et al,^103^ 2017	China	January 2003 to September 2011	Cross-sectional	68	Patients aged 18-35 y with CRC surgical resections at Department of General Surgery, Nanfang Hospital Southern Medical University	Moderate	Time to diagnosis (claims and medical records)
Zhu et al,^104^ 2015	China	January 1996 to December 2013	Cross-sectional	83	Patients aged 13-30 y with CRC at Shanghai Changzheng Hospital	Low	Symptoms at presentation (claims and medical records) Time to diagnosis (claims and medical records)

Abbreviations: CRC, colorectal cancer; *ICD-9-CM*, *International Classification of Diseases, Ninth Revision, Clinical Modification*; *ICD-O-3*, *International Classification of Diseases for Oncology, Third Revision*; IDA, iron deficiency anemia; NIS, National Inpatient Sample; NR, not reported; VHA, Veterans Health Administration.

### Presenting Signs and Symptoms

**Figure 2.**
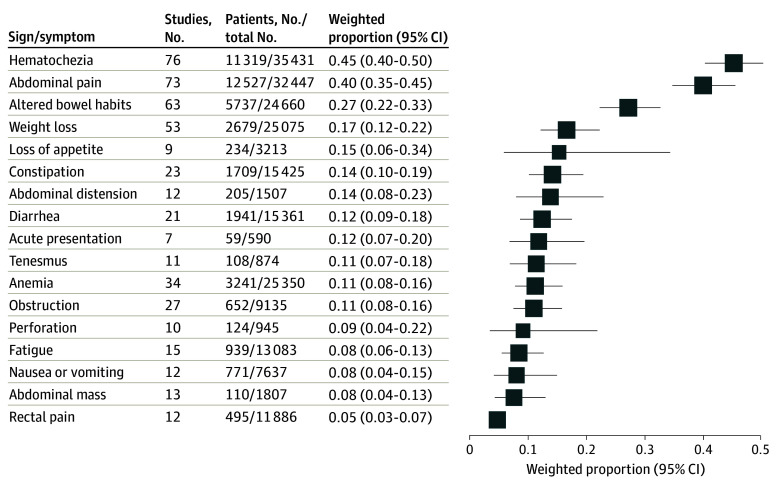
Pooled Proportions of Presenting Signs and Symptoms for Early-Onset Colorectal Cancer

There were 78 studies^12,13,18,31,32,33,34,35,36,37,38,39,40,41,42,43,44,45,46,47,48,49,50,51,52,53,54,55,56,57,58,59,60,61,62,63,64,65,66,67,68,69,70,71,72,73,74,75,76,77,78,79,80,81,82,83,84,85,86,87,88,89,90,91,92,94,95,96,97,98,99,100,101,102,103,104,105,106,107,108^ that reported on 17 signs and symptoms at presentation, based on claims or medical records (66 studies),^12,13,27,28,30,32,33,34,38,39,40,42,43,44,45,47,48,51,52,53,54,55,56,57,58,59,60,61,62,63,64,65,66,67,68,69,70,71,72,73,74,75,76,77,79,81,83,84,86,87,88,89,90,91,92,94,95,96,97,98,99,100,101,102,103,104^ patient report (6 studies),^18,29,37,46,80,82^ or other (7 studies).^31,35,41,49,50,78,85^ (Figure 2; eFigure 1 in Supplement 1). In adults with EOCRC, the 3 most common presenting signs and symptoms were hematochezia (pooled prevalence, 45% [95% CI, 40%-50%]; 76 studies),^12,13,18,27,28,29,30,31,33,34,35,37,38,39,40,41,42,43,44,45,46,47,48,49,50,51,52,53,54,55,56,57,58,59,60,61,62,63,65,66,67,68,69,70,71,72,73,74,75,76,77,78,79,80,81,82,83,84,85,86,87,88,89,90,91,92,94,95,96,97,98,99,100,101,102,104^ abdominal pain (pooled prevalence, 40% [95% CI, 35%-45%]; 73 studies),^12,13,18,27,28,29,30,31,33,34,35,37,38,39,40,42,43,44,45,46,47,48,49,50,51,52,53,54,55,56,57,58,59,60,61,62,63,64,65,66,67,70,71,72,73,74,75,76,77,78,79,80,81,82,83,84,85,87,88,89,90,91,92,94,95,96,97,98,99,100,101,102,104^ and altered bowel habits, which included constipation, diarrhea, alternating bowel habits, or alternating diarrhea or constipation (pooled prevalence, 27% [95% CI, 22%-33%]; 63 studies).^12,18,28,29,30,31,33,34,35,37,38,39,43,44,45,46,47,48,49,50,51,52,53,54,55,56,57,58,59,60,61,62,63,64,65,66,67,68,70,71,72,74,75,76,77,78,79,80,83,84,85,87,88,90,92,94,95,96,98,99,100,102,104^

When evaluating patterns by geography, the 3 most common presenting signs and symptoms were the same in both the US (20 studies)^12,33,34,39,44,47,59,61,68,69,70,74,81,86,88,92,94,95,97,98^ and non-US (58 studies)^13,27,28,29,35,37,38,40,41,42,43,45,46,48,49,50,51,52,53,54,55,56,57,58,60,62,63,64,65,66,67,71,72,73,75,76,77,78,79,80,82,83,84,85,87,89,90,91,96,99,100,101,102,104^ studies (eFigure 2 in Supplement 1). When stratifying by age of study population, there were 42 studies^12,18,28,29,30,32,33,34,38,39,40,42,44,45,48,50,54,61,62,67,69,70,71,74,75,78,80,82,86,87,88,89,90,91,92,94,95,96,97,98,99,100^ including adults aged 50 years or younger and 25 studies^31,35,37,41,43,46,47,49,51,53,55,56,59,60,63,64,65,66,68,72,77,81,83,84,85^ including adults aged 40 years and younger. In both groups, the top 3 presenting signs and symptoms were consistent with the primary results (eFigure 3 in Supplement 1). Primary results were unchanged in studies with low risk of bias; although in studies with moderate risk of bias, the 3 most common presenting signs and symptoms varied: hematochezia (pooled prevalence, 43% [95% CI, 34%-53%]; 9 studies), abdominal pain (pooled prevalence, 36% [95% CI, 26%-48%]; 9 studies) and obstruction (pooled prevalence, 24% [95% CI. 16%-33%]; 2 studies) (eFigure 4 in Supplement 1). When examining data source used to ascertain presenting sign or symptom, only studies with a poorly defined data source showed alternative most common presenting symptoms: loss of appetite (pooled prevalence, 58% [95% CI, 40%-74%]; 2 studies), hematochezia (pooled prevalence, 57% [95% CI, 37%-75%]; 7 studies), and abdominal pain (pooled prevalence, 54% [95% CI, 36%-71%]; 6 studies) (eFigure 5 in Supplement 1). Meta-regression analyses by percentage of male study participants or year of study publication across the 17 signs and symptoms for CRC were not found to account for a significant amount of between-study heterogeneity.

### Associations of Signs and Symptoms With EOCRC Risk

**Figure 3.**
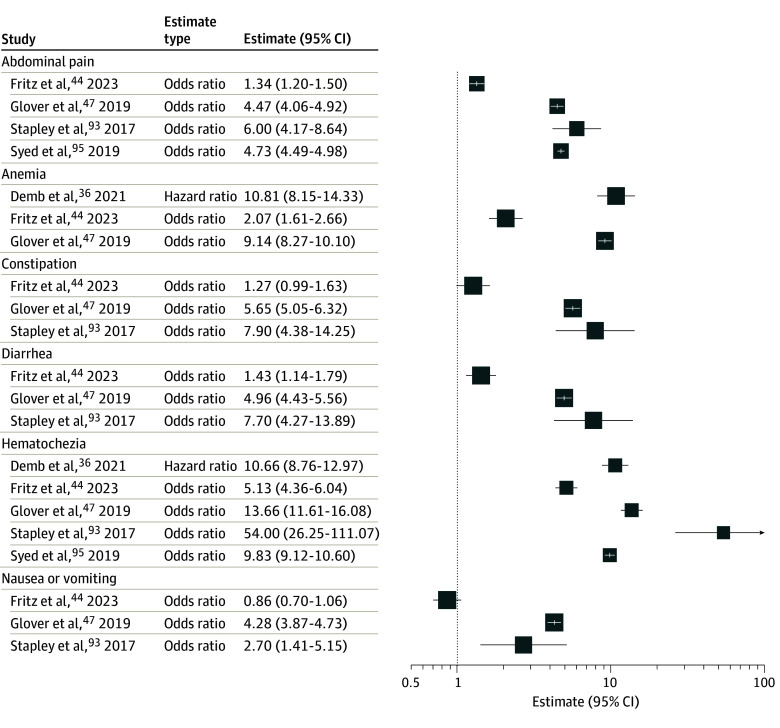
Association Between Symptoms and the Risk of Early-Onset Colorectal Cancer Size of box indicates number of estimates.

There were 5 studies^36,44,47,93,95^ examining the association of EOCRC risk with abdominal pain, anemia, constipation, diarrhea, hematochezia, and nausea or vomiting (Figure 3). Hematochezia (relative estimate range, 5.2-54.0; 5 studies),^36,44,47,93,95^ abdominal pain (relative estimate range, 1.3-6.0; 4 studies),^44,47,93,95^ and anemia (relative estimate range, 2.1-10.8; 3 studies)^36,44,47^ were associated with higher likelihood of CRC compared with no CRC.

### Time From Symptom Onset to Diagnosis

**Figure 4.**
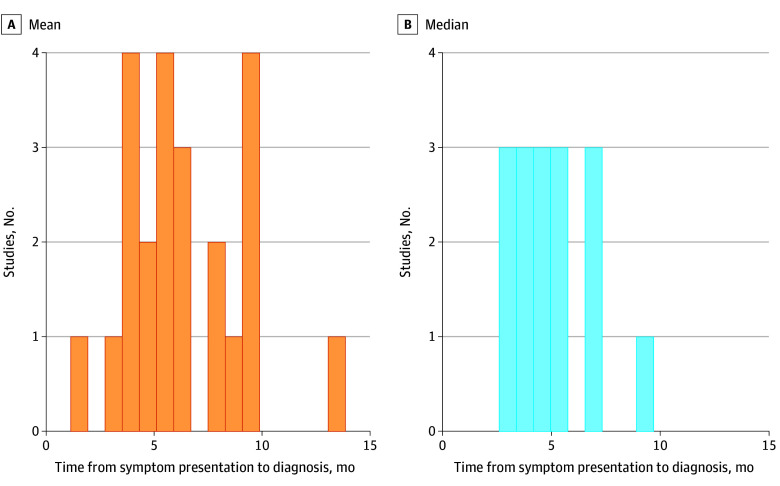
Histogram of Study Frequencies of Time From Symptom Onset to Diagnosis, by Measurement Type

There were 34 studies^18,28,29,31,32,33,34,37,38,41,43,44,50,52,53,54,56,57,58,61,62,71,75,80,81,83,84,85,86,88,90,94,96,104^ that reported a continuous measure of time from sign or symptom presentation to diagnosis, with 23 studies providing a mean result and 16 studies providing a median result (eTable 3 in Supplement 1). The time from symptom onset to EOCRC diagnosis was reported as a mean (range) of 6.4 (1.8-13.7) months and a median (range) of 4.1 (2.0-8.7) months (Figure 4). When classifying time from sign or symptom onset to diagnosis by measurement type (medical record, patient reported, not well defined), there was considerable heterogeneity. When excluding studies with inadequately defined data sources, the time from symptom onset to EOCRC diagnosis was a mean (range) of 6.6 (3.0-13.7) months and median (range) of 3.8 (2.0-8.7) months (eFigure 6 in Supplement 1).

## Discussion

In this systematic review and meta-analysis, nearly half of individuals diagnosed with EOCRC presented with hematochezia and abdominal pain, which were associated with 5- to 54-fold and 1.3- to 6-fold increased likelihood of CRC, respectively. An interval of 4 to 6 months from symptom onset to EOCRC diagnosis was common. These findings underscore the need for clinicians to consider EOCRC as part of the differential diagnosis for patients presenting with potential red flag signs and symptoms, and to follow up through either confirmation of diagnosis and sign or symptom resolution when a benign cause is suspected, or colonoscopy referral to rule out CRC based on sign or symptom severity or absence of diagnosis or sign or symptom resolution after initial workup and management for a suspected benign cause.

Our finding that 45% of individuals with EOCRC presented with hematochezia aligns with current clinical paradigms—hematochezia (or rectal bleeding) is often cited as a common presenting symptom among patients with CRC.^105^ In addition, the 5 studies^36,44,47,93,95^ that measured the association between hematochezia and EOCRC risk found estimates between 5.1 and 54.0, underscoring the urgent need for these patients to undergo comprehensive diagnostic evaluation. A full colonoscopy should be pursued when individuals younger than 50 years present with hematochezia, according to guidelines from the American Society for Gastrointestinal Endoscopy and European Panel on the Appropriateness of Gastrointestinal Endoscopy.^106,107^ A high index of suspicion for CRC in younger patients with hematochezia may be particularly useful to identify patients with high risk, given the high frequency and association with CRC.

Our review also found that nearly half of individuals with EOCRC reported abdominal pain, based on evidence from 73 studies^12,13,18,27,28,29,30,31,33,34,35,37,38,39,40,42,43,44,45,46,47,48,49,50,51,52,53,54,55,56,57,58,59,60,61,62,63,64,65,66,67,70,71,72,73,74,75,76,77,78,79,80,81,82,83,84,85,87,88,89,90,91,92,94,95,96,97,98,99,100,101,102,104^ and a 1.3- to 6-fold positive association with EOCRC risk across 4 studies.^44,47,93,95^ Given its association with a myriad of gastrointestinal conditions, the American Academy of Family Physicians recommends computed tomography for evaluating patients with acute right or left lower quadrant abdominal pain and ultrasonography for right upper quadrant pain, though the guidelines also recommend identifying associated symptoms to better focus a differential diagnosis.^108^ It may be inefficient and unrealistic to perform colonoscopy for all adults younger than 45 years with isolated abdominal pain, given the low diagnostic yield^109^ and insufficient capacity across the US to accommodate this group. Nevertheless, the fact that 40% of patients with EOCRC presented with abdominal pain and 27% presented with altered bowel habits reinforces that any new symptom should be comprehensively evaluated by a clinician. Our findings suggest that EOCRC should be part of the initial differential diagnosis, and that a plan for follow-up should be in place, such as a 30- to 60-day follow-up visit to confirm whether the original working diagnosis was correct, the red flag sign or symptom has resolved, or to refer for colonoscopy to exclude EOCRC if these criteria are not met.^110^ We postulate that all benign causes of red flag signs or symptoms either can be diagnostically confirmed or should resolve with initial treatment. When an alternative diagnosis is not confirmed or signs or symptoms fail to resolve, a colonoscopy to rule out EOCRC should be pursued. Abdominal pain could serve as a marker to prompt further patient-clinician discussion about additional medical history, which could help determine whether further diagnostic work-up is warranted.

Globally and in the US, hematochezia, abdominal pain, and altered bowel habits were the 3 most common signs and symptoms. The fourth most common symptom differed based on geographic location—diarrhea among US studies and loss of appetite in non-US studies. The findings highlight how nonspecific symptoms are frequently present at EOCRC diagnosis and emphasize the need for medical professionals to be aware of the symptoms most associated with EOCRC, to refine clinical practice pathways and minimize late EOCRC detection.

The mean time from sign or symptom onset to EOCRC diagnosis was found to be 6.4 months (median, 4 months). A recent study using administrative claims data in Canada from 2003 to 2018 reported the greatest delay occurring between the first investigation and diagnosis (78 days) with short turnaround times between presentation and first investigation (5 days) or diagnosis and treatment start (23 days). Date of first presentation was defined by the physician visit related to the diagnostic examination (endoscopy, surgery, or imaging).^111^ The data are mixed on whether decreasing time to diagnosis would improve outcomes, but it is well established that risk for progression to more advanced-stage disease increases over time. Another claims-based study from Canada found that young individuals with CRC had longer diagnostic intervals compared with middle-aged patients, although young patients with metastatic EOCRC had a short diagnostic interval, likely due to more noticeable or concerning symptoms.^32^ Other studies found that differences between older and younger patients with CRC in stage at presentation were not just associated with delayed diagnosis, but could be associated with additional biological and genetic factors.^33^

Nevertheless, it is prudent to address potential physician and patient barriers to timely workup. Younger patients may experience ongoing signs and symptoms and delay seeking medical attention.^88^ Potential reasons for these delays include a patient believing they are too young to worry about cancer or a lack of access to primary care or health insurance.^88,110^ Clarifying how these signs and symptoms are associated with EOCRC could give patients greater urgency to report these symptoms sooner, leading to quicker diagnostic workup and resolution. For clinicians, particularly those in primary care, recognition of clues and appropriate diagnostic workup for concerning signs and symptoms is paramount to early EOCRC detection. However, prior studies found that clinicians often dismiss these signs and symptoms or misattribute them to more benign conditions, such as attributing rectal bleeding to hemorrhoids, without conducting further diagnostic evaluation.^15,112^ This can leave a potentially concerning sign or symptom unresolved for an extended period of time, and for some patients, delay EOCRC diagnosis. To avoid missing an EOCRC diagnosis, clinicians should work with patients to ensure concerning signs and symptoms undergo diagnostic evaluation to identify and resolve the underlying cause.

Our study has several strengths. Our approach distilled a tremendous amount of global data over several decades into clear and practical information that is immediately useful to clinicians. We applied strict study selection criteria to capture only individuals younger than 50 years with nonhereditary CRC to represent an individual with average risk diagnosed with EOCRC beginning in 1996, when EOCRC rates started to increase. The meta-analysis adjusted for or stratified by potential contributors to study heterogeneity, including study quality, age of study population, country of study origin, percentage of male study participants, and year of publication.

### Limitations

Our study has some limitations. There was significant heterogeneity across studies, which impacted our ability to meta-analyze some of our results. This was most significant in assessment of the associations of signs and symptoms with EOCRC, where a lack of a consistent comparator group hindered our ability to pool estimates for the associations. Additionally, we were unable to compare EOCRC risk against other potential outcomes, which might have better contextualized the relative risk. In our measurement of association of signs and symptoms with EOCRC, studies did not measure the potential likelihood of reverse causation—whether EOCRC was associated with sign or symptom presentation. We were unable to evaluate the impact of time to diagnosis on CRC outcomes due to a limited number of studies answering this question. In addition, sign- and symptom-based data extracted from studies used in this review were often extracted cross-sectionally to characterize patients with EOCRC at study baseline, limiting our access to stratified or more granular results by age, sex, race and ethnicity, or genetic ancestry, which could have better contextualized the burden of signs and symptoms and relevant EOCRC risk. We were unable to examine the constellation of signs and symptoms since we lacked individual-level data from each study and could not provide a positive predictive value for symptoms. However, we anticipate patients may have presented with multiple signs and symptoms and encourage clinicians to consider the full list of common presenting signs and symptoms and their prevalence to aid in EOCRC risk assessment.

## Conclusions

This systematic review and meta-analysis of studies examining sign and symptom presentation of EOCRC found that hematochezia, abdominal pain, altered bowel habits, and unexplained weight loss were the most common presenting signs and symptoms in patients diagnosed with EOCRC. Markedly increased EOCRC risk was seen in adults with hematochezia and abdominal pain. Furthermore, time from sign or symptom presentation to EOCRC diagnosis was often between 4 and 6 months. These findings and the increasing risk of CRC in individuals younger than 50 years highlight the urgent need to educate clinicians and patients about these signs and symptoms to ensure that diagnostic workup and resolution are not delayed. Adapting current clinical practice to identify and address these signs and symptoms through careful clinical triage and follow-up could help limit morbidity and mortality associated with EOCRC.

## Notes

Supplement 1. **eTable 1.** Search Strategy **eTable 2.** Risk of Bias Assessment Using the Joanna Briggs Institute Critical Appraisal Checklist Tool **eTable 3.** Time From Symptom Presentation to Diagnosis Measurement Across Studies
eMethods.
**eFigure 1.** Forest Plots of Proportions of Presenting Signs and Symptoms for EOCRC, by Sign or Symptom **eFigure 2.** Pooled Proportions of Presenting Signs and Symptoms for EOCRC by Geography **eFigure 3.** Pooled Proportions of Presenting Signs and Symptoms for EOCRC; Stratified Analysis by Age Group **eFigure 4.** Pooled Proportions of Presenting Signs and Symptoms for EOCRC, Stratified Analysis by Risk of Bias **eFigure 5.** Pooled Proportions of Presenting Signs and Symptoms for EOCRC, Stratified Analysis by Data Source **eFigure 6.** Histograms of Mean and Median Diagnosis Stratified by Data Source

Supplement 2.
Data Sharing Statement
