# Supplementary material for: Red Flag Signs and Symptoms for Patients With Early-Onset Colorectal Cancer: A Systematic Review and Meta-Analysis
Source: JAMA Netw Open. 2024 May 24;7(5):e2413157. doi: 10.1001/jamanetworkopen.2024.13157 (PMC11127127; doi:10.1001/jamanetworkopen.2024.13157)
Supplement: Supplement 1. — eTable 1. Search Strategy eTable 2. Risk of Bias Assessment Using the Joanna Briggs Institute Critical Appraisal Checklist Tool eTable 3. Time From Symptom Presentation to Diagnosis Measurement Across Studies eMethods. eFigure 1. Forest Plots of Proportions of Presenting Signs and Symptoms for EOCRC, by Sign or Symptom eFigure 2. Pooled Proportions of Presenting Signs and Symptoms for EOCRC by Geography eFigure 3. Pooled Proportions of Presenting Signs and Symptoms for EOCRC; Stratified Analysis by Age Group eFigure 4. Pooled Proportions of Presenting Signs and Symptoms for EOCRC, Stratified Analysis by Risk of Bias eFigure 5. Pooled Proportions of Presenting Signs and Symptoms for EOCRC, Stratified Analysis by Data Source eFigure 6. Histograms of Mean and Median Diagnosis Stratified by Data Source [file jamanetwopen-e2413157-s001.pdf]

Supplemental Online Content

Demb J, Kolb JM, Dounel J, et al. Red flag signs and symptoms for patients with early-onset colorectal cancer: a systematic review and meta-analysis. *JAMA Netw Open*. 2024;7(5):e2413157. doi:10.1001/jamanetworkopen.2024.13157

eTable 1. Search Strategy
eTable 2. Risk of Bias Assessment Using the Joanna Briggs Institute Critical Appraisal Checklist Tool
eTable 3. Time From Symptom Presentation to Diagnosis Measurement Across Studies
eMethods.
eFigure 1. Forest Plots of Proportions of Presenting Signs and Symptoms for EOCRC, by Sign or Symptom
eFigure 2. Pooled Proportions of Presenting Signs and Symptoms for EOCRC by Geography
eFigure 3. Pooled Proportions of Presenting Signs and Symptoms for EOCRC; Stratified Analysis by Age Group
eFigure 4. Pooled Proportions of Presenting Signs and Symptoms for EOCRC, Stratified Analysis by Risk of Bias
eFigure 5. Pooled Proportions of Presenting Signs and Symptoms for EOCRC, Stratified Analysis by Data Source
eFigure 6. Histograms of Mean and Median Diagnosis Stratified by Data Source

This supplemental material has been provided by the authors to give readers additional information about their work.

© 2024 Demb J et al. *JAMA Network Open.*

eTable 1. Search Strategy

**PubMed**		**Embase**		**CINAHL**		**Web of Science**	
**Early onset terms**							
Early onset[tiab]	OR	'onset age'/exp	OR	TI Early onset OR AB Early onset	OR	"early onset"	OR
young onset[tiab]	OR	‘early onset’	OR	TI young onset OR AB young onset	OR	"young onset"	OR
age of onset[MeSH]	OR	‘young onset’	OR	(MH "Age of Onset")	OR	age NEAR/2 onset	OR
age onset[tiab]	OR	‘age onset’:ti,ab	OR	TI age onset OR AB age onset	OR	"young* patient*"	OR
"younger patient*"[tiab]	OR	'young* patient*'	OR	"young* patient*"	OR	((adolescent* OR "young adult*") AND colo*)	
"young patient*"[tiab]	OR	((adolescent:ti,ab OR 'young adult*':ti,ab) AND colo*:ti,ab)	OR	((TI ((adolescent* OR young adult*)) OR AB ((adolescent* OR young adult*))) AND (TI colo* OR AB colo*))	OR		
((adolescent*[tiab] OR young adult*[tiab]) AND colo*[tiab])	OR	('young adult'/exp AND 'colorectal cancer'/exp)		((MH "Young Adult") AND (MH "Colorectal Neoplasms+"))			
(Young Adult[Mesh] AND "Colorectal Neoplasms"[MeSH])							
**Colorectal cancer terms**							
Colorectal Neoplasms[MeSH]	OR	'colon cancer'/exp	OR	(MH "Colorectal Neoplasms+")	OR	"colorectal cancer"	OR
"colorectal cancer"[tiab]	OR	'rectum cancer'/exp	OR	"colorectal cancer"	OR	"colorectal adenocarcinoma"	OR
"colorectal adenocarcinoma"[tiab]	OR	‘colon cancer’	OR	"colorectal adenocarcinoma"	OR	"colon cancer"	OR
"colon cancer"[tiab]	OR	‘colon neoplasms’	OR	"colon cancer"	OR	"rectal cancer"	
"rectal cancer"[tiab]		‘colorectal neoplasms’	OR	"rectal cancer"			
		‘colorectal cancer’	OR				
		‘rectal cancer’	OR				
		‘rectal neoplasms’					
**Detection, diagnosis, or symptoms**							
recognition[tiab]	OR	'early cancer diagnosis'/exp	OR	(MH "Diagnosis+")	OR	diagnos*	OR
diagnos*[tiab]	OR	'cancer diagnosis'/exp	OR	TI recognition OR AB recognition	OR	recogni*	OR
"diagnosis"[MeSH Terms]	OR	(recognition):ab,ti	OR	TI diagnos* OR diagnos* diagnos*	OR	"risk factors"	OR
detect*[tiab]	OR	(diagnosis):ab,ti	OR	(MH "Early Detection of Cancer")	OR	detect*	OR
sign*[tiab]	OR	(detection):ab,ti	OR	(MH "Risk Factors")	OR	sign*	OR

© 2024 Demb J et al. *JAMA Network Open.*

symptom*[tiab]	OR	(signs):ab,ti	OR	TI detect* OR AB detect*	OR	symptom* NEAR/2 onset	OR
(symptom*[tiab] AND onset[tiab])	OR	(symptoms):ab,ti	OR	TI sign* OR AB sign*	OR	"clinical presentation"	
clinical presentation[tiab]	OR	‘symptom onset’	OR	TI symptom* OR AB symptom*	OR		
"Early Detection of Cancer"[MeSH]	OR	‘clinical presentation’	OR	TI (symptom* AND onset) OR AB (symptom* AND onset)	OR		
Risk Factors[Mesh]	OR	‘Early Detection of Cancer’	OR	TI clinical presentation OR AB clinical presentation	OR		
risk*[tiab]		'early cancer'/exp		TI risk* OR AB risk*			
Search Strategies Combined							
((Early onset[tiab] OR young onset[tiab] OR age of onset[MeSH] OR age onset[tiab] OR "younger patient*"[tiab] OR "young patient*"[tiab] OR ((adolescent*[tiab] OR young adult*[tiab]) AND colo*[tiab]) OR (Young Adult[Mesh] AND "Colorectal Neoplasms"[MeSH])) AND (Colorectal Neoplasms[MeSH] OR "colorectal cancer"[tiab] OR "colorectal adenocarcinoma"[tiab] OR "colon cancer"[tiab] OR "rectal cancer"[tiab])) AND (recognition[tiab] OR diagnos*[tiab] OR "diagnosis"[MeSH Terms] OR detect*[tiab] OR sign*[tiab] OR symptom*[tiab] OR (symptom*[tiab] AND onset[tiab]) OR clinical presentation[tiab] OR		('onset age'/exp OR 'early onset' OR 'young onset' OR 'age onset':ti,ab OR 'young* patient*' OR ((adolescent:ti,ab OR 'young adult*':ti,ab) AND colo*:ti,ab) OR ('young adult'/exp AND 'colorectal cancer'/exp)) AND ('colon cancer'/exp OR 'rectum cancer'/exp OR 'colon cancer' OR 'colon neoplasms' OR 'colorectal neoplasms' OR 'colorectal cancer' OR 'rectal cancer' OR 'rectal neoplasms') AND ('early cancer diagnosis'/exp OR 'cancer diagnosis'/exp OR recognition:ab,ti OR diagnosis:ab,ti OR detection:ab,ti OR signs:ab,ti OR symptoms:ab,ti OR 'symptom onset' OR 'clinical presentation' OR 'early detection of cancer' OR 'early cancer'/exp)		(TI Early onset OR AB Early onset OR TI young onset OR AB young onset OR (MH "Age of Onset") OR TI age onset OR AB age onset OR "young* patient*" OR ((TI ((adolescent* OR young adult*)) OR AB ((adolescent* OR young adult*))) AND (TI colo* OR AB colo*)) OR ((MH "Young Adult") AND (MH "Colorectal Neoplasms+"))) AND ((MH "Colorectal Neoplasms+") OR "colorectal cancer" OR "colorectal adenocarcinoma" OR "colon cancer" OR "rectal cancer") AND ((MH "Diagnosis+") OR TI recognition OR AB recognition OR TI diagnos* OR diagnos* diagnos* OR (MH "Early Detection of Cancer") OR (MH "Risk Factors") OR TI detect* OR AB detect* OR TI sign* OR AB sign* OR TI symptom* OR AB symptom* OR TI (symptom* AND onset) OR AB (symptom* AND onset) OR TI clinical presentation OR AB clinical presentation OR TI risk* OR AB risk*)		("early onset" OR "young onset" OR age NEAR/2 onset OR "young* patient*" OR ((adolescent* OR "young adult*") AND colo*)) AND ("colorectal cancer" OR "colorectal adenocarcinoma" OR "colon cancer" OR "rectal cancer") AND (diagnos* OR recogni* OR "risk factors" OR detect* OR sign* OR symptom* NEAR/2 onset OR "clinical presentation")	

© 2024 Demb J et al. *JAMA Network Open.*

"Early Detection of Cancer"[MeSH] OR Risk Factors[Mesh] OR risk*[tiab])							

© 2024 Demb J et al. *JAMA Network Open.*

eTable 2. Risk of Bias Assessment Using the Joanna Briggs Institute Critical Appraisal Checklist Tool

A. Cohort studies

Source	1	2	3	4	5	6	7	8	9	10	11	Risk of Bias
Demb et al,[36] 2021	Yes	Yes	Yes	Yes	Yes	Yes	Yes	Yes	No	Yes	Yes	Low

JBI Critical Appraisal Checklist for Cohort Studies:

1) Were the two groups similar and recruited from the same population?
2) Were the exposures measured similarly to assign people to both exposed and unexposed groups?
3) Was the exposure measured in a valid and reliable way?
4) Were confounding factors identified?
5) Were strategies to deal with confounding factors stated?
6) Were the groups/participants free of the outcome at the start of the study (or at the moment of exposure)?
7) Were the outcomes measured in a valid and reliable way?
8) Was the follow up time reported and sufficient to be long enough for outcomes to occur?
9) Was follow up complete, and if not, were the reasons to loss to follow up described and explored?
10) Were strategies to address incomplete follow up utilized?
11) Was appropriate statistical analysis used?

Risk of bias:

Low: >75% Yes to JBI Questions
Mod: 50-75% Yes to JBI Questions
High: <50% Yes to JBI Questions

© 2024 Demb J et al. *JAMA Network Open.*

B. Cross-Sectional Studies

Source	1	2	3	4	5	6	7	8	Risk of Bias
Al-Barrak et al,[27] 2011	Yes	Yes	Yes	Yes	N/A	N/A	N/A	Yes	Low
Arhi et al,[28] *2019*	Yes	Yes	Yes	Yes	N/A	N/A	Yes	Yes	Low
Arriba et al,[29] *2019*	Yes	Yes	No	No	N/A	N/A	No	Yes	Moderate
Avellaneda et al,[30] *2021*	Yes	Yes	Yes	Yes	N/A	N/A	N/A	Yes	Low
Ben-Ishay et al,[18] *2013*	Yes	Yes	No	No	N/A	N/A	No	Yes	Moderate
Bouassida et al,[31] *2012*	Yes	Yes	No	No	N/A	N/A	No	Yes	Moderate
Castelo et al,[32] *2023*	Yes	Yes	Yes	Yes	N/A	N/A	Yes	Yes	Low
Cercek et al,[12] *2021*	Yes	Yes	Yes	Yes	N/A	N/A	N/A	Yes	Low
Chen et al,[33] *2017*	Yes	Yes	Yes	Yes	N/A	N/A	No	Yes	Low
Chiu et al,[34] *2023*	Yes	Yes	Yes	Yes	N/A	N/A	No	Yes	Low
Chou et al,[35] *2011*	Yes	Yes	No	No	N/A	N/A	N/A	Yes	Moderate
De Silva et al,[37] *2000*	Yes	Yes	No	No	N/A	N/A	No	Yes	Moderate
De Sousa et al,[38] *2014*	Yes	Yes	Yes	Yes	N/A	N/A	No	Yes	Low
Dharwadkar et al,[39] *2021*	Yes	Yes	Yes	Yes	N/A	N/A	N/A	Yes	Low
Di Leo et al,[40] *2021*	Yes	Yes	Yes	Yes	N/A	N/A	Yes	Yes	Low
El-Hennawy et al,[41] *2003*	No	Yes	No	No	N/A	N/A	No	Yes	High
Fayaz et al,[42] *2018*	Yes	Yes	Yes	Yes	N/A	N/A	N/A	Yes	Low
Foppa et al,[43] *2021*	Yes	Yes	Yes	Yes	N/A	N/A	Yes	Yes	Low
Frostberg et al,[45] *2020*	Yes	Yes	Yes	Yes	N/A	N/A	N/A	Yes	Low
Ganapathi et al,[46] *2011*	Yes	Yes	No	No	N/A	N/A	N/A	Yes	Moderate
Goh et al,[48] *2020*	Yes	Yes	Yes	Yes	N/A	N/A	N/A	Yes	Low
Gul et al,[49] *2012*	No	Yes	No	No	N/A	N/A	N/A	Yes	High
Gunel et al,[50] *2001*	Yes	Yes	No	No	N/A	N/A	No	Yes	Moderate
Haleshappa et al,[51] *2017*	Yes	Yes	Yes	Yes	N/A	N/A	N/A	Yes	Low
Haresh et al,[52] *2016*	Yes	Yes	Yes	Yes	N/A	N/A	Yes	Yes	Low
Haroon et al,[53] *2013*	Yes	Yes	Yes	Yes	N/A	N/A	Yes	Yes	Low
Jarrar et al,[54] *2022*	Yes	Yes	Yes	Yes	N/A	N/A	Yes	Yes	Low
Josifovski et al,[55] *2004*	Yes	Yes	Yes	Yes	N/A	N/A	N/A	Yes	Low
Kansakar et al,[56] *2012*	Yes	Yes	Yes	Yes	N/A	N/A	Yes	Yes	Low
Kaplan et al,[57] *2013*	Yes	Yes	Yes	Yes	N/A	N/A	Yes	Yes	Low
Kaplan et al,[58] *2019*	Yes	Yes	Yes	Yes	N/A	N/A	Yes	Yes	Low
Karsten et al,[59] *2008*	No	Yes	Yes	Yes	N/A	N/A	N/A	Yes	Low
Kocian et al,[60] *2017*	Yes	Yes	Yes	Yes	N/A	N/A	N/A	Yes	Low
Lapumnuaypol et al,[61] *2018*	Yes	Yes	Yes	Yes	N/A	N/A	Yes	Yes	Low
Law et al,[62] *2017*	Yes	Yes	Yes	Yes	N/A	N/A	Yes	Yes	Low
Leff et al,[63] *2007*	Yes	Yes	Yes	Yes	N/A	N/A	N/A	Yes	Low
Leopa et al,[64] *2023*	Yes	Yes	Yes	Yes	N/A	N/A	Yes	Yes	Low
Limaiem et al,[65] *2018*	No	Yes	Yes	Yes	N/A	N/A	N/A	Yes	Low
Lin et al,[66] *2005*	Yes	Yes	Yes	Yes	N/A	N/A	Yes	Yes	Low

© 2024 Demb J et al. *JAMA Network Open.*

Makmun et al,[67] 2021	Yes	Yes	Yes	Yes	N/A	N/A	N/A	Yes	Low
Melendez-Rosado et al,[68] 2022	Yes	Yes	Yes	Yes	N/A	N/A	N/A	Yes	Low
Mogor et al,[69] 2019	Yes	Yes	Yes	Yes	N/A	N/A	N/A	Yes	Low
Myers et al,[70] 2013	Yes	Yes	Yes	Yes	N/A	N/A	N/A	Yes	Low
Nagai et al,[71] 2016	Yes	Yes	Yes	Yes	N/A	N/A	Yes	Yes	Low
Nikolic et al,[72] 2023	Yes	Yes	Yes	Yes	N/A	N/A	N/A	Yes	Low
Ozaydin et al,[73] 2019	Yes	Yes	Yes	Yes	N/A	N/A	N/A	Yes	Low
Park et al,[74] 2022	Yes	Yes	Yes	Yes	N/A	N/A	N/A	Yes	Low
Patel et al,[75] 2016	Yes	Yes	Yes	Yes	N/A	N/A	Yes	Yes	Low
Plunkett et al,[76] 2014	Yes	Yes	Yes	Yes	N/A	N/A	N/A	Yes	Low
Poudyal et al,[77] 2017	Yes	Yes	Yes	Yes	N/A	N/A	N/A	Yes	Low
Quach et al,[78] 2012	No	Yes	No	No	N/A	N/A	No	Yes	High
Rajagopalan et al,[79] 2021	Yes	Yes	Yes	Yes	N/A	N/A	N/A	Yes	Low
Raman et al,[80] 2014	Yes	Yes	No	No	N/A	N/A	No	Yes	Moderate
Reddy et al,[81] 2021	Yes	Yes	Yes	Yes	N/A	N/A	Yes	Yes	Low
Rho et al,[13] 2017	Yes	Yes	Yes	Yes	N/A	N/A	N/A	Yes	Low
Riaz et al,[82] 2017	No	No	No	No	N/A	N/A	N/A	Yes	High
Ruiz et al,[83] 2016	Yes	Yes	Yes	Yes	N/A	N/A	Yes	Yes	Low
Saidi et al,[84] 2018	No	Yes	Yes	Yes	N/A	N/A	Yes	Yes	Low
Saluja et al,[85] 2014	Yes	Yes	No	No	N/A	N/A	No	Yes	Moderate
Sandhu et al,[86] 2020	Yes	Yes	Yes	Yes	N/A	N/A	Yes	Yes	Low
Schellerer et al,[87] 2012	Yes	Yes	Yes	Yes	N/A	N/A	N/A	Yes	Low
Scott et al,[88] 2016	Yes	Yes	Yes	Yes	N/A	N/A	Yes	Yes	Low
Silva et al,[89] 2019	Yes	Yes	Yes	Yes	N/A	N/A	N/A	Yes	Low
Silva et al,[90] 2020	Yes	Yes	Yes	Yes	N/A	N/A	Yes	Yes	Low
Singh et al,[91] 2020	Yes	Yes	Yes	Yes	N/A	N/A	N/A	Yes	Low
Skalitsky et al,[92] 2023	Yes	Yes	Yes	Yes	N/A	N/A	Yes	Yes	Low
Strum et al,[94] 2019	Yes	Yes	Yes	Yes	N/A	N/A	Yes	Yes	Low
Trivedi et al,[96] 2022	Yes	Yes	Yes	Yes	N/A	N/A	Yes	Yes	Low
Vajrevelu et al,[97] 2021	Yes	Yes	Yes	Yes	N/A	N/A	N/A	Yes	Low
Vakil et al,[98] 2021	Yes	Yes	Yes	Yes	N/A	N/A	N/A	Yes	Low
Wan lbrahim et al,[99] 2020	Yes	Yes	Yes	Yes	N/A	N/A	N/A	Yes	Low
Wong et al,[100] 2021	Yes	Yes	Yes	Yes	N/A	N/A	N/A	Yes	Low
Zahir et al,[101] 2014	Yes	Yes	Yes	Yes	N/A	N/A	N/A	Yes	Low
Zhang et al,[102] 2009	Yes	Yes	Yes	Yes	N/A	N/A	N/A	Yes	Low
Zhao et al,[103] 2017	Yes	Yes	No	No	N/A	N/A	N/A	Yes	Moderate
Zhu et al,[104] 2015	Yes	Yes	Yes	Yes	N/A	N/A	N/A	Yes	Low

JBI Critical Appraisal Checklist for Cross-Sectional Studies:

1) Were the criteria for inclusion in the sample clearly defined?
2) Were the study subjects and the setting described in detail?
3) Were the [symptoms at presentation] measured in a valid and reliable way?
4) Were objective, standard criteria used for measurement of the condition?

© 2024 Demb J et al. *JAMA Network Open.*

5) Were confounding factors identified? (Not relevant for measurement of proportion of symptoms or time to diagnosis)
6) Were strategies to deal with confounding factors stated? (Not relevant for measurement of proportion of symptoms or time to diagnosis)
7) Was the [time to diagnosis] measured in a valid and reliable way?
8) Was appropriate statistical analysis used?

© 2024 Demb J et al. *JAMA Network Open.*

C. Case-Control Studies

Source	1	2	3	4	5	6	7	8	9	10	Risk of Bias
Fritz et al,[44] 2023	Yes	Yes	Yes	Yes	Yes	Yes	Yes	Yes	Yes	Yes	Low
Glover et al,[47] 2019	Yes	Yes	Yes	Yes	Yes	Yes	Yes	Yes	Yes	Yes	Low
Stapley et al,[93] 2017	Yes	Yes	Yes	Yes	Yes	Yes	Yes	Yes	Yes	Yes	Low
Syed et al,[95] 2019	Yes	Yes	Yes	Yes	Yes	Yes	Yes	Yes	Yes	Yes	Low

JBI Critical Appraisal Checklist for Case Control Studies

1) Were the groups comparable other than the presence of disease in cases or the absence of disease in controls?
2) Were cases and controls matched appropriately?
3) Were the same criteria used for identification of cases and controls?
4) Was exposure measured in a standard, valid and reliable way?
5) Was exposure measured in the same way for cases and controls?
6) Were confounding factors identified?
7) Were strategies to deal with confounding factors stated?
8) Were outcomes assessed in a standard, valid and reliable way for cases and controls?
9) Was the exposure period of interest long enough to be meaningful?
10) Was appropriate statistical analysis used?

Risk of bias:
Low: >75% Yes to JBI Questions
Mod: 50-75% Yes to JBI Questions
High: <50% Yes to JBI Questions

© 2024 Demb J et al. *JAMA Network Open.*

eTable 3. Time from symptom presentation to diagnosis measurement across studies (n=39)

Study	Continuous Time to Diagnosis (in months)	Categorical Time to Diagnosis
Arhi et al,[28] 2019	Median (IQR): 3.6 (2-7.5)	
Arriba et al,[29] 2019	Mean ± SD: 5.59 ± 8.59	
Ben-Ishay et al,[18] 2013	Mean: 5.3	
Bouassida et al,[31] 2012	Mean: 1.8	
Castelo et al,[32] 2023	Mean ± SD: 4.03 ± 3.9<br>Median (IQR): 2.6 (0.9-62)	
Chen et al,[33] 2017	Mean ± SD: 8.1 ± 15.5<br>Median (IQR): 4.27 (2-8.8)	
Chiu et al,[34] 2023	Median (Range): 2 (0.5-6)	
De Silva et al,[37] 2000	Mean (Range): 4.2 (2-7)	
De Sousa et al,[38] 2014	Mean ± SD: 6.3 ± 4	
Di Leo et al,[40] 2021		0-1 months: 10/54<br>2-5 months: 12/54<br>6-12 months: 18/54<br>>12 months: 14/54
El-Hennawy et al,[41] 2003	Mean ± SD: 9.8 ± 2.6	
Foppa et al,[43] 2021	Mean ± SD: 13.7 ± 9.8	
Fritz et al,[44] 2023	Median (IQR): 8.7 (4.8-15.9)	
Gunel et al,[50] 2001	Mean: 4	
Haresh et al,[52] 2016	Median (Range): 6 (1-60)	
Haroon et al,[53] 2013	Mean ± SD: 9.7 ± 6	
Jarrar et al,[54] 2022	Median (Range): 5 (1-18)	
Kansakar et al,[56] 2012	Mean: 7.8 months (1999-2003);<br>Mean: 5.6 months (2004-2008)	
Kaplan et al,[57] 2013	Median (Range): 3 (0-48)	
Kaplan et al,[58] 2019	Median (Range): 3 (0-48)	
Lapumnuaypol et al,[61] 2018	Mean ± SD: 3 ± 2.9	
Law et al,[62] 2017	Median (Range): 2 (0.1-60)	
Leopa et al,[64] 2023		3 months: 1/19<br>6 months: 3/19<br>9 months: 10/19<br>12 months: 5/19
Nagai et al,[71] 2016	Mean ± SD: 5.2 ± 5.4	
Patel et al,[75] 2016	Median: 3.8	

© 2024 Demb J et al. *JAMA Network Open.*

Quach et al,[78] 2012		<1 month: 12/112<br>1-3 months: 53/122<br>3-6 months: 20/112<br>6-12 months: 19/112<br>>12 months: 8/112
Raman et al,[80] 2014	Median: 3	
Reddy et al,[81] 2021	Mean ± SD: 4.5 ± 11.4	
Ruiz et al,[83] 2016	Mean (Range): 8.3 (0.5-48)	
Saidi et al,[84] 2018	Mean ± SD: 6.15 ± 7.5	
Saluja et al,[85] 2014	Median (Range): 6 (1-48)	
Sandhu et al,[86] 2020	Mean ± SD: 9.83 ± 10.9<br>Median (IQR): 6 (2-12)	
Scott et al,[88] 2016	Median: 4.37	
Silva et al,[90] 2020	Mean ± SD: 4.2 ± 4.6	
Skalitsky et al,[92] 2023		<1 month: 49/286<br>1-3 months: 79/286<br>>3 months: 131/286<br>Not available: 27/286
Strum et al,[94] 2019	Mean (Range): 6.7 (0.1-36)<br>Median (Range): 5 (0.1-36)	
Trivedi et al,[96] 2022	Mean ± SD: 9.36	<3 months: 21/148<br>3-6 months: 67/148<br>6-12 months: 44/148<br>>12 months: 16/148
Zhao et al,[103] 2017		≤6 months: 37/68<br>>6 months: 31/68
Zhu et al,[104] 2015	Mean (Range): 4.6 (0-24)	

© 2024 Demb J et al. *JAMA Network Open.*

eMethods.

Code Syntax

1. **R syntax used to derive Hematochezia forest plot and weighted proportion Question 1**

Note: To use this code, data need to be in proportion format, with a known X and known N (noted as HEMATO_X and HEMATO_N) below.

```
#metaprop function with HK method
HEMATO_1<-metaprop(HEMATO_X, HEMATO_N, studlab=`Study ID`, data=HEMATO,
random=TRUE, method.random.ci="HK")

#Aggregating estimates into a table
HEMATO_1.random.est1 <- c(symptom="Hematochezia", estimate=HEMATO_1$TE.random,
ci.lb=HEMATO_1$lower.random, ci.ub=HEMATO_1$upper.random,
sei=HEMATO_1$seTE.random, count=sum(nrow(HEMATO)),
cases=sum(HEMATO$HEMATO_X), sample=sum(HEMATO$HEMATO_N))

#Create and export forest plot
pdf(file = "HEMATO_ForestPlot.pdf", width = 8, height = 14)
forest(HEMATO_1, fontsize = 8, spacing = .75, common = FALSE, xlim=c(0,1))
dev.off()
```

2. **R syntax used to derive aggregate forest plot.**

Note: The code used above is applied to each sign and symptom and then aggregated to be entered into a single plot with the code below.

```
#Merging all of the estimates into a single data frame
TOTALS1<-rbind(IDA_1.random.est1, HEMATO_1.random.est1, UWL_1.random.est1,
FAT_1.random.est1, CIBH_1.random.est1, CONS_1.random.est1, DIAR_1.random.est1,
MASS_1.random.est1, APAIN_1.random.est1, LOA_1.random.est1, TENE_1.random.est1,
ABDIS_1.random.est1, NVOM_1.random.est1, OBS_1.random.est1, PERF_1.random.est1,
ACUTE_1.random.est1, REC_1.random.est1)
TOTALS1<-as.data.frame(TOTALS1)

#Converting all variables in the above table into numeric values
TOTALS1$estimate<-as.numeric(TOTALS1$estimate)
TOTALS1$ci.lb<-as.numeric(TOTALS1$ci.lb)
TOTALS1$ci.ub<-as.numeric(TOTALS1$ci.ub)
TOTALS1$sei<-as.numeric(TOTALS1$sei)
TOTALS1$proportion<-paste0(TOTALS1$cases, "/", TOTALS1$sample)

#Creating aggregated forest plot
OVERALL<-metagen(estimate, sei, studlab=symptom, data=TOTALS1, random=TRUE,
method.random.ci="HK", sm="PLOGIT")
```

© 2024 Demb J et al. *JAMA Network Open.*

```
pdf(file = "Overall_ForestPlot.pdf", width = 11, height = 8)
forest(OVERALL, sortvar=-estimate, just="center", just.studlab="left", overall=FALSE,
overall.hetstat=FALSE, xlim=c(0,0.6), weight.study="random", layout="JAMA",
leftcols=c("studlab", "count", "proportion", "effect.ci"),leftlabs = c("Sign/Symptom", "Number \nof
Studies", "Proportion", "Weighted \nProportion (95% CI)"), col.square="skyblue2", xlab="Weighted
Proportion (95% CI)")
dev.off()
```

3. R syntax used to develop forest plot of Hematochezia effect estimates in Question 2.

Note: Estimates need to be entered in log form with a calculated standard error.

```
#Code used to bring effect estimates into single table.
HEMATO2_1<-metagen(logOR, SE, data=HEMATO2,random=TRUE, method.random.ci="HK",
sm="OR")

#Forest plot code removing overall pooled estimates and overall heterogeneity estimates
forest(HEMATO2_1, overall=FALSE, overall.hetstat=FALSE, weight.study="random",
layout="JAMA", xlim=c(1, 100), at=c(1,5,10,50,100), leftcols=c("StudyID2", "effect.ci"),
leftlabs=c("Study (Author, Year)", "OR (95% CI)"))

#Used to export estimates from metagen function above in a table, if needed.
HEMATO2_1.random.est1 <- c(symptom="Hematochezia", estimate=HEMATO2_1$TE.random,
ci.lb=HEMATO2_1$lower.random, ci.ub=HEMATO2_1$upper.random,
sei=HEMATO2_1$seTE.random)
print(HEMATO2_1.random.est1)
```

4. R syntax used to create histograms of time to diagnosis data for Question 3.

Note: Data need to be entered with either mean or median estimates (or both) and classified as such. T2D_EST in the code below is either mean or median estimates, while T2D_TYPE is the indicator of whether that estimate is a mean or media.

```
#Base ggplot2 code for the histogram
AIM3_PLOT<-ggplot(AIM3_2, aes(x = T2D_EST, fill = T2D_TYPE)) + geom_histogram(bins=20)
+ facet_grid(. ~ T2D_TYPE)

#Customization code to clean up the plot
AIM3_PLOT+theme_bw(base_size=18)+theme(legend.position="")+theme(panel.spacing=unit(1,"li
nes"))+theme(panel.grid.major = element_blank(), panel.grid.minor =
element_blank())+ylab("Number of Studies")+xlab("Time from Symptom Presentation to Diagnosis
(Months)")+scale_x_continuous(expand = c(0, 0), breaks = seq(0, 15, 5), lim = c(0,
15))+scale_y_continuous(expand = c(0, 0), lim=c(0,5))
```

© 2024 Demb J et al. *JAMA Network Open.*

eFigure 1. Forest plots of proportions of presenting signs and symptoms for EOCRC, by sign or symptom.

A. Hematochezia

Study	Events	Total	Proportion	95%−CI
Al−Barrak 2011	32	62	0.52	[0.39; 0.65]
Arhi 2019	71	508	0.14	[0.11; 0.17]
Arriba 2019	38	98	0.39	[0.29; 0.49]
Avellaneda 2021	1	32	0.03	[0.00; 0.16]
Ben−Ishay 2013	8	31	0.26	[0.12; 0.45]
Bouassida 2012	9	40	0.23	[0.11; 0.38]
Cercek 2021	311	759	0.41	[0.37; 0.45]
Chen 2017	104	253	0.41	[0.35; 0.47]
Chiu 2023	66	103	0.64	[0.54; 0.73]
Chou 2011	31	69	0.45	[0.33; 0.57]
deSilva 2000	19	60	0.32	[0.20; 0.45]
deSousa 2014	51	66	0.77	[0.65; 0.87]
Dharwadkar 2021	123	319	0.39	[0.33; 0.44]
DiLeo 2021	19	54	0.35	[0.23; 0.49]
El−Hennawy 2003	25	26	0.96	[0.80; 1.00]
Fayaz 2018	36	130	0.28	[0.20; 0.36]
Foppa 2021	64	101	0.63	[0.53; 0.73]
Fritz 2023	363	5075	0.07	[0.06; 0.08]
Frostberg 2020	80	174	0.46	[0.38; 0.54]
Ganapathi 2011	31	59	0.53	[0.39; 0.66]
Glover 2019	160	1680	0.10	[0.08; 0.11]
Goh 2020	21	65	0.32	[0.21; 0.45]
Gul 2012	33	50	0.66	[0.51; 0.79]
Gunel 2001	39	100	0.39	[0.29; 0.49]
Haleshappa 2017	43	89	0.48	[0.38; 0.59]
Haresh 2016	46	60	0.77	[0.64; 0.87]
Haroon 2013	22	23	0.96	[0.78; 1.00]
Jarrar 2022	33	67	0.49	[0.37; 0.62]
Josifovski 2004	5	19	0.26	[0.09; 0.51]
Kansakar 2012	33	62	0.53	[0.40; 0.66]
Kaplan 2013	20	56	0.36	[0.23; 0.50]
Kaplan 2019	53	141	0.38	[0.30; 0.46]
Karsten 2008	14	41	0.34	[0.20; 0.51]
Kocian 2017	18	38	0.47	[0.31; 0.64]
Lapumnuaypol 2018	42	109	0.39	[0.29; 0.48]
Law 2017	69	154	0.45	[0.37; 0.53]
Leff 2007	28	49	0.57	[0.42; 0.71]
Limaiem 2018	16	32	0.50	[0.32; 0.68]
Lin 2005	11	45	0.24	[0.13; 0.40]
Makmun 2021	99	205	0.48	[0.41; 0.55]
Melendez−Rosado 2022	39	56	0.70	[0.56; 0.81]
Mogor 2019	962	2748	0.35	[0.33; 0.37]
Myers 2013	99	180	0.55	[0.47; 0.62]
Nagai 2016	28	70	0.40	[0.28; 0.52]
Nikolic 2023	28	87	0.32	[0.23; 0.43]
Ozaydin 2019	16	32	0.50	[0.32; 0.68]
Park 2022	1840	3856	0.48	[0.46; 0.49]
Patel 2016	13	18	0.72	[0.47; 0.90]
Plunkett 2014	14	50	0.28	[0.16; 0.42]
Poudyal 2017	13	30	0.43	[0.25; 0.63]
Quach 2012	58	112	0.52	[0.42; 0.61]
Rajagopalan 2021	41	75	0.55	[0.43; 0.66]
Raman 2014	46	72	0.64	[0.52; 0.75]
Reddy 2021	63	139	0.45	[0.37; 0.54]
Rho 2017	100	224	0.45	[0.38; 0.51]
Riaz 2017	59	105	0.56	[0.46; 0.66]
Ruiz 2016	132	196	0.67	[0.60; 0.74]
Saidi 2018	38	70	0.54	[0.42; 0.66]
Saluja 2014	44	66	0.67	[0.54; 0.78]
Sandhu 2020	88	173	0.51	[0.43; 0.59]
Schellerer 2012	146	244	0.60	[0.53; 0.66]
Scott 2016	34	56	0.61	[0.47; 0.74]
Silva 2019	224	781	0.29	[0.26; 0.32]
Silva 2020	12	39	0.31	[0.17; 0.48]
Singh 2020	5	22	0.23	[0.08; 0.45]
Skalitzky 2023	152	286	0.53	[0.47; 0.59]
Strum 2019	67	109	0.61	[0.52; 0.71]
Syed 2019	790	5710	0.14	[0.13; 0.15]
Trivedi 2022	133	148	0.90	[0.84; 0.94]
Vajravelu 2021	2835	6163	0.46	[0.45; 0.47]
Vakil 2021	239	637	0.38	[0.34; 0.41]
WanIbrahim 2020	216	893	0.24	[0.21; 0.27]
Wong 2021	60	178	0.34	[0.27; 0.41]
Zahir 2014	68	131	0.52	[0.43; 0.61]
Zhang 2009	275	488	0.56	[0.52; 0.61]
Zhu 2015	55	83	0.66	[0.55; 0.76]
**Random effects model**		**35431**	**0.45**	**[0.40; 0.50]**

Heterogeneity: $I^2 = 98\%$, $\tau^2 = 0.7345$, $p = 0$

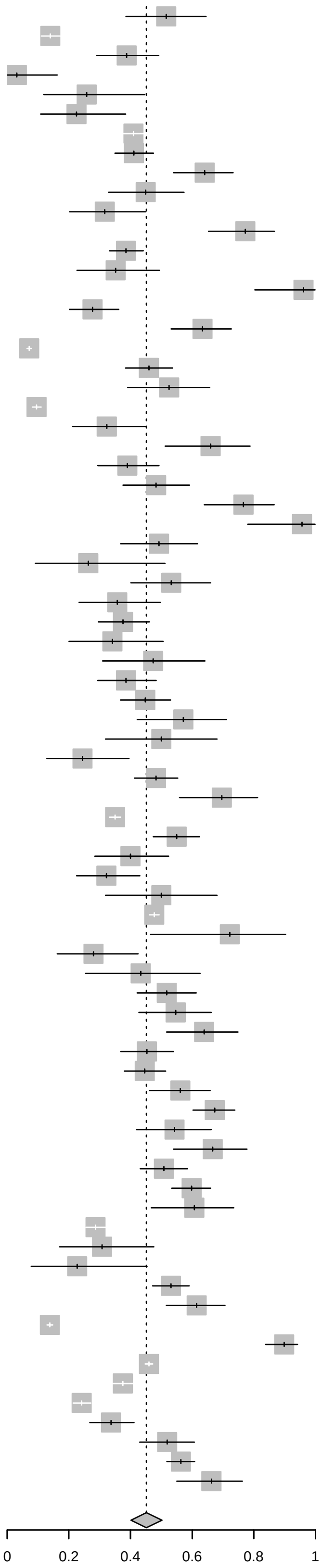

© 2024 Demb J et al. *JAMA Network Open.*

B. Abdominal Pain

Study	Events	Total	Proportion	95%−CI
Al−Barrak 2011	31	62	0.50	[0.37; 0.63]
Arhi 2019	107	508	0.21	[0.18; 0.25]
Arriba 2019	24	98	0.24	[0.16; 0.34]
Avellaneda 2021	6	32	0.19	[0.07; 0.36]
Ben−Ishay 2013	20	31	0.65	[0.45; 0.81]
Bouassida 2012	21	40	0.53	[0.36; 0.68]
Cercek 2021	263	759	0.35	[0.31; 0.38]
Chen 2017	78	253	0.31	[0.25; 0.37]
Chiu 2023	66	103	0.64	[0.54; 0.73]
Chou 2011	27	69	0.39	[0.28; 0.52]
deSilva 2000	11	60	0.18	[0.10; 0.30]
deSousa 2014	49	66	0.74	[0.62; 0.84]
Dharwadkar 2021	77	319	0.24	[0.20; 0.29]
DiLeo 2021	2	54	0.04	[0.00; 0.13]
Fayaz 2018	96	130	0.74	[0.65; 0.81]
Foppa 2021	5	101	0.05	[0.02; 0.11]
Fritz 2023	588	5075	0.12	[0.11; 0.12]
Frostberg 2020	99	174	0.57	[0.49; 0.64]
Ganapathi 2011	21	59	0.36	[0.24; 0.49]
Glover 2019	890	1680	0.53	[0.51; 0.55]
Goh 2020	24	65	0.37	[0.25; 0.50]
Gul 2012	42	50	0.84	[0.71; 0.93]
Gunel 2001	22	100	0.22	[0.14; 0.31]
Haleshappa 2017	41	89	0.46	[0.35; 0.57]
Haresh 2016	24	60	0.40	[0.28; 0.53]
Haroon 2013	5	23	0.22	[0.07; 0.44]
Jarrar 2022	41	67	0.61	[0.49; 0.73]
Josifovski 2004	6	19	0.32	[0.13; 0.57]
Kansakar 2012	27	62	0.44	[0.31; 0.57]
Kaplan 2013	23	56	0.41	[0.28; 0.55]
Kaplan 2019	56	141	0.40	[0.32; 0.48]
Karsten 2008	14	41	0.34	[0.20; 0.51]
Kocian 2017	7	38	0.18	[0.08; 0.34]
Lapumnuaypol 2018	43	109	0.39	[0.30; 0.49]
Law 2017	94	154	0.61	[0.53; 0.69]
Leff 2007	17	49	0.35	[0.22; 0.50]
Leopa 2023	2	19	0.11	[0.01; 0.33]
Limaiem 2018	15	32	0.47	[0.29; 0.65]
Lin 2005	24	45	0.53	[0.38; 0.68]
Makmun 2021	114	205	0.56	[0.49; 0.63]
Myers 2013	54	180	0.30	[0.23; 0.37]
Nagai 2016	7	70	0.10	[0.04; 0.20]
Nikolic 2023	16	87	0.18	[0.11; 0.28]
Ozaydin 2019	12	32	0.38	[0.21; 0.56]
Park 2022	1277	3856	0.33	[0.32; 0.35]
Patel 2016	8	18	0.44	[0.22; 0.69]
Plunkett 2014	35	50	0.70	[0.55; 0.82]
Poudyal 2017	26	30	0.87	[0.69; 0.96]
Quach 2012	71	112	0.63	[0.54; 0.72]
Rajagopalan 2021	43	75	0.57	[0.45; 0.69]
Raman 2014	16	72	0.22	[0.13; 0.34]
Reddy 2021	50	139	0.36	[0.28; 0.45]
Rho 2017	126	224	0.56	[0.49; 0.63]
Riaz 2017	17	105	0.16	[0.10; 0.25]
Ruiz 2016	133	196	0.68	[0.61; 0.74]
Saidi 2018	50	70	0.71	[0.59; 0.82]
Saluja 2014	41	66	0.62	[0.49; 0.74]
Schellerer 2012	56	244	0.23	[0.18; 0.29]
Scott 2016	4	56	0.07	[0.02; 0.17]
Silva 2019	309	781	0.40	[0.36; 0.43]
Silva 2020	22	39	0.56	[0.40; 0.72]
Singh 2020	18	22	0.82	[0.60; 0.95]
Skalitzky 2023	134	286	0.47	[0.41; 0.53]
Strum 2019	29	109	0.27	[0.19; 0.36]
Syed 2019	3060	5710	0.54	[0.52; 0.55]
Trivedi 2022	54	148	0.36	[0.29; 0.45]
Vajravelu 2021	2897	6163	0.47	[0.46; 0.48]
Vakil 2021	238	637	0.37	[0.34; 0.41]
WanIbrahim 2020	266	893	0.30	[0.27; 0.33]
Wong 2021	56	178	0.31	[0.25; 0.39]
Zahir 2014	114	131	0.87	[0.80; 0.92]
Zhang 2009	131	488	0.27	[0.23; 0.31]
Zhu 2015	35	83	0.42	[0.31; 0.54]
**Random effects model**		**32447**	**0.40**	**[0.35; 0.46]**

Heterogeneity: $I^2 = 98\%$, $\tau^2 = 0.8880$, $p = 0$

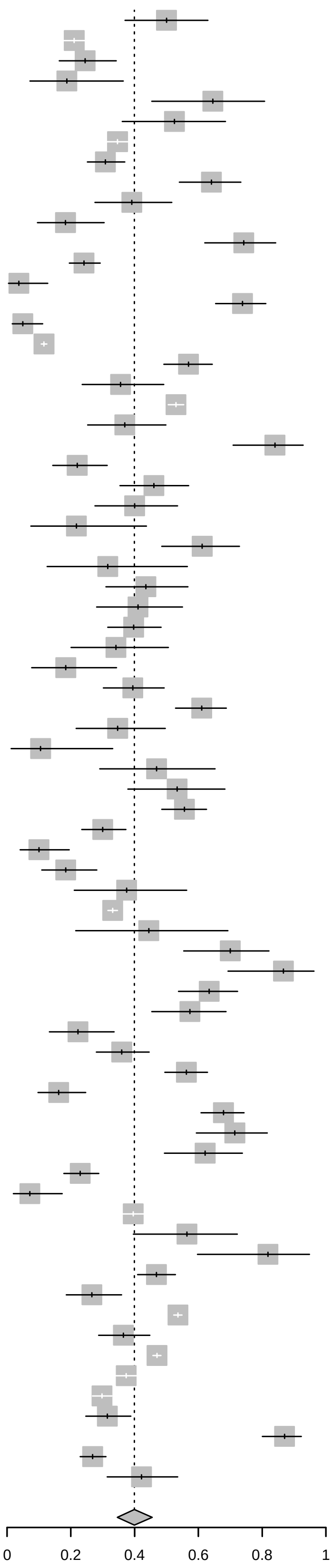

© 2024 Demb J et al. *JAMA Network Open.*

C. Altered Bowel Habits

Study	Events	Total	Proportion	95%−CI
Arhi 2019	19	508	0.04	[0.02; 0.06]
Arriba 2019	22	98	0.22	[0.15; 0.32]
Avellaneda 2021	10	32	0.31	[0.16; 0.50]
Ben−Ishay 2013	16	31	0.52	[0.33; 0.70]
Bouassida 2012	7	40	0.17	[0.07; 0.33]
Cercek 2021	150	759	0.20	[0.17; 0.23]
Chen 2017	3	253	0.01	[0.00; 0.03]
Chiu 2023	41	103	0.40	[0.30; 0.50]
Chou 2011	10	69	0.14	[0.07; 0.25]
deSilva 2000	12	60	0.20	[0.11; 0.32]
deSousa 2014	42	66	0.64	[0.51; 0.75]
Dharwadkar 2021	23	319	0.07	[0.05; 0.11]
Foppa 2021	17	101	0.17	[0.10; 0.26]
Fritz 2023	246	5075	0.05	[0.04; 0.05]
Frostberg 2020	89	174	0.51	[0.43; 0.59]
Ganapathi 2011	15	59	0.25	[0.15; 0.38]
Glover 2019	800	1680	0.48	[0.45; 0.50]
Goh 2020	13	65	0.20	[0.11; 0.32]
Gul 2012	43	50	0.86	[0.73; 0.94]
Gunel 2001	14	100	0.14	[0.08; 0.22]
Haleshappa 2017	37	89	0.42	[0.31; 0.53]
Haresh 2016	25	60	0.42	[0.29; 0.55]
Haroon 2013	12	23	0.52	[0.31; 0.73]
Jarrar 2022	18	67	0.27	[0.17; 0.39]
Josifovski 2004	3	19	0.16	[0.03; 0.40]
Kansakar 2012	17	62	0.27	[0.17; 0.40]
Kaplan 2013	22	56	0.39	[0.26; 0.53]
Kaplan 2019	37	141	0.26	[0.19; 0.34]
Karsten 2008	4	41	0.10	[0.03; 0.23]
Kocian 2017	8	38	0.21	[0.10; 0.37]
Lapumnuaypol 2018	13	109	0.12	[0.07; 0.20]
Law 2017	86	154	0.56	[0.48; 0.64]
Leff 2007	25	49	0.51	[0.36; 0.66]
Leopa 2023	9	19	0.47	[0.24; 0.71]
Limaiem 2018	17	32	0.53	[0.35; 0.71]
Lin 2005	6	45	0.13	[0.05; 0.27]
Makmun 2021	115	205	0.56	[0.49; 0.63]
Melendez−Rosado 2022	11	56	0.20	[0.10; 0.32]
Myers 2013	37	180	0.21	[0.15; 0.27]
Nagai 2016	6	70	0.09	[0.03; 0.18]
Nikolic 2023	21	87	0.24	[0.16; 0.35]
Park 2022	953	3856	0.25	[0.23; 0.26]
Patel 2016	12	18	0.67	[0.41; 0.87]
Plunkett 2014	10	50	0.20	[0.10; 0.34]
Poudyal 2017	11	30	0.37	[0.20; 0.56]
Quach 2012	11	112	0.10	[0.05; 0.17]
Rajagopalan 2021	42	75	0.56	[0.44; 0.67]
Raman 2014	9	72	0.12	[0.06; 0.22]
Ruiz 2016	56	196	0.29	[0.22; 0.35]
Saidi 2018	45	70	0.64	[0.52; 0.75]
Saluja 2014	35	66	0.53	[0.40; 0.65]
Schellerer 2012	71	244	0.29	[0.23; 0.35]
Scott 2016	8	56	0.14	[0.06; 0.26]
Silva 2020	10	39	0.26	[0.13; 0.42]
Skalitzky 2023	153	286	0.53	[0.48; 0.59]
Strum 2019	5	109	0.05	[0.02; 0.10]
Syed 2019	1440	5710	0.25	[0.24; 0.26]
Trivedi 2022	42	148	0.28	[0.21; 0.36]
Vakil 2021	191	637	0.30	[0.26; 0.34]
WanIbrahim 2020	278	893	0.31	[0.28; 0.34]
Wong 2021	84	178	0.47	[0.40; 0.55]
Zhang 2009	105	488	0.22	[0.18; 0.25]
Zhu 2015	45	83	0.54	[0.43; 0.65]
**Random effects model**		**24660**	**0.27**	**[0.22; 0.33]**

Heterogeneity: $I^2 = 97\%$, $\tau^2 = 1.0504$, $p = 0$

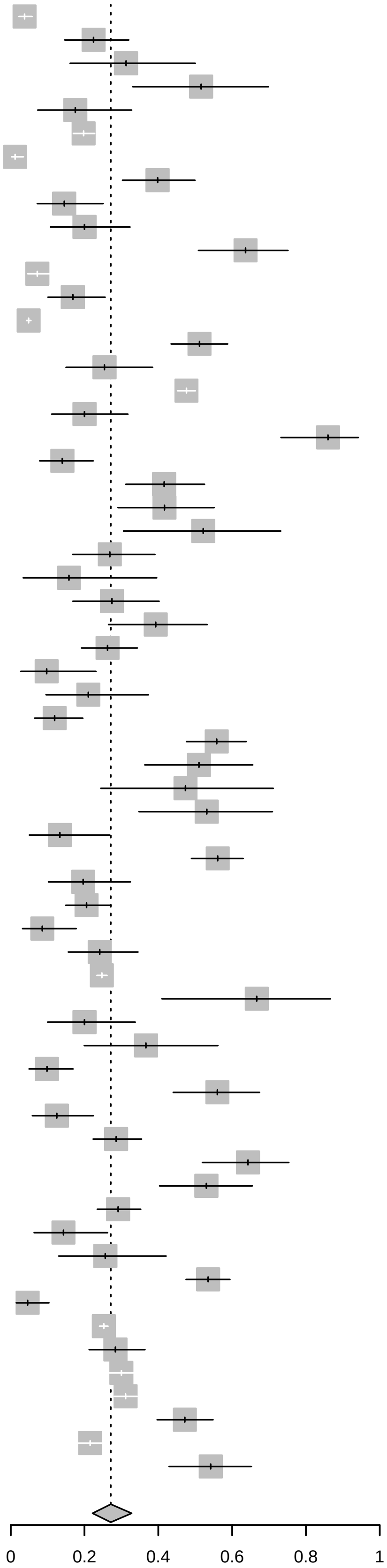

© 2024 Demb J et al. *JAMA Network Open.*

D. Weight Loss

Study	Events	Total	Proportion	95%−CI
Arhi 2019	6	508	0.01	[0.00; 0.03]
Arriba 2019	21	98	0.21	[0.14; 0.31]
Ben−Ishay 2013	9	31	0.29	[0.14; 0.48]
Cercek 2021	52	759	0.07	[0.05; 0.09]
Chen 2017	6	253	0.02	[0.01; 0.05]
Chiu 2023	14	103	0.14	[0.08; 0.22]
Chou 2011	8	69	0.12	[0.05; 0.22]
deSilva 2000	3	60	0.05	[0.01; 0.14]
deSousa 2014	42	66	0.64	[0.51; 0.75]
DiLeo 2021	5	54	0.09	[0.03; 0.20]
Fayaz 2018	22	130	0.17	[0.11; 0.24]
Foppa 2021	1	101	0.01	[0.00; 0.05]
Frostberg 2020	49	174	0.28	[0.22; 0.35]
Ganapathi 2011	1	59	0.02	[0.00; 0.09]
Glover 2019	120	1680	0.07	[0.06; 0.08]
Gul 2012	42	50	0.84	[0.71; 0.93]
Haleshappa 2017	24	89	0.27	[0.18; 0.37]
Haroon 2013	5	23	0.22	[0.07; 0.44]
Kaplan 2013	11	56	0.20	[0.10; 0.32]
Kaplan 2019	17	141	0.12	[0.07; 0.19]
Karsten 2008	4	41	0.10	[0.03; 0.23]
Kocian 2017	1	38	0.03	[0.00; 0.14]
Lapumnuaypol 2018	17	102	0.17	[0.10; 0.25]
Law 2017	46	154	0.30	[0.23; 0.38]
Leff 2007	4	49	0.08	[0.02; 0.20]
Leopa 2023	4	19	0.21	[0.06; 0.46]
Limaiem 2018	1	32	0.03	[0.00; 0.16]
Makmun 2021	72	205	0.35	[0.29; 0.42]
Melendez−Rosado 2022	12	56	0.21	[0.12; 0.34]
Myers 2013	20	180	0.11	[0.07; 0.17]
Nikolic 2023	6	87	0.07	[0.03; 0.14]
Park 2022	174	3856	0.05	[0.04; 0.05]
Patel 2016	1	18	0.06	[0.00; 0.27]
Plunkett 2014	17	50	0.34	[0.21; 0.49]
Poudyal 2017	23	30	0.77	[0.58; 0.90]
Quach 2012	46	112	0.41	[0.32; 0.51]
Rajagopalan 2021	21	75	0.28	[0.18; 0.40]
Reddy 2021	24	139	0.17	[0.11; 0.25]
Rho 2017	79	224	0.35	[0.29; 0.42]
Ruiz 2016	103	196	0.53	[0.45; 0.60]
Saidi 2018	10	70	0.14	[0.07; 0.25]
Saluja 2014	40	66	0.61	[0.48; 0.72]
Silva 2019	52	781	0.07	[0.05; 0.09]
Silva 2020	19	39	0.49	[0.32; 0.65]
Singh 2020	4	22	0.18	[0.05; 0.40]
Skalitzky 2023	91	286	0.32	[0.26; 0.38]
Syed 2019	490	5710	0.09	[0.08; 0.09]
Vajravelu 2021	493	6163	0.08	[0.07; 0.09]
WanIbrahim 2020	163	893	0.18	[0.16; 0.21]
Wong 2021	51	178	0.29	[0.22; 0.36]
Zahir 2014	86	131	0.66	[0.57; 0.74]
Zhang 2009	15	488	0.03	[0.02; 0.05]
Zhu 2015	32	81	0.40	[0.29; 0.51]
**Random effects model**		**25075**	**0.17**	**[0.12; 0.22]**

Heterogeneity: $I^2 = 97\%$, $\tau^2 = 1.6694$, $p = 0$

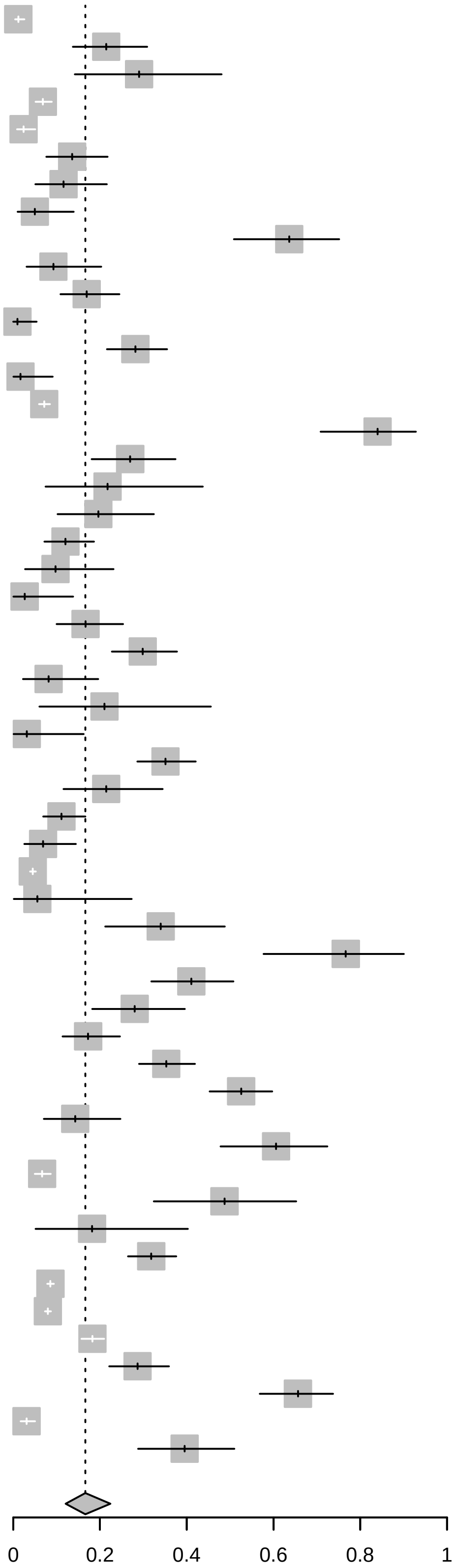

© 2024 Demb J et al. *JAMA Network Open.*

E. Loss of Appetite

Study	Events	Total	Proportion	95%−CI
deSilva 2000	3	60	0.05	[0.01; 0.14]
Glover 2019	50	1680	0.03	[0.02; 0.04]
Gul 2012	35	50	0.70	[0.55; 0.82]
Lapumnuaypol 2018	6	109	0.06	[0.02; 0.12]
Poudyal 2017	20	30	0.67	[0.47; 0.83]
Saluja 2014	30	66	0.45	[0.33; 0.58]
Silva 2020	3	39	0.08	[0.02; 0.21]
Skalitzky 2023	34	286	0.12	[0.08; 0.16]
WanIbrahim 2020	53	893	0.06	[0.04; 0.08]
**Random effects model**		**3213**	**0.15**	**[0.05; 0.39]**

Heterogeneity: $I^2 = 98\%$, $\tau^2 = 2.4893$, $p < 0.01$

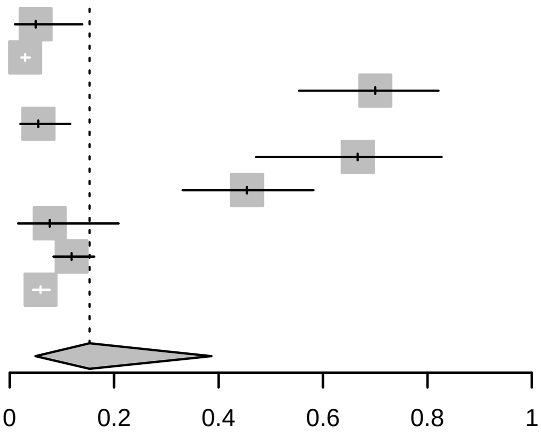

© 2024 Demb J et al. *JAMA Network Open.*

F. Constipation

Study	Events	Total	Proportion	95%−CI
Al−Barrak 2011	7	62	0.11	[0.05; 0.22]
Arhi 2019	24	508	0.05	[0.03; 0.07]
Ben−Ishay 2013	9	31	0.29	[0.14; 0.48]
Bouassida 2012	7	40	0.17	[0.07; 0.33]
Chen 2017	12	253	0.05	[0.02; 0.08]
Chou 2011	6	69	0.09	[0.03; 0.18]
DiLeo 2021	1	54	0.02	[0.00; 0.10]
Fayaz 2018	20	130	0.15	[0.10; 0.23]
Fritz 2023	106	5075	0.02	[0.02; 0.03]
Glover 2019	410	1680	0.24	[0.22; 0.27]
Jarrar 2022	22	67	0.33	[0.22; 0.45]
Lapumnuaypol 2018	6	109	0.06	[0.02; 0.12]
Law 2017	49	154	0.32	[0.25; 0.40]
Leopa 2023	4	19	0.21	[0.06; 0.46]
Makmun 2021	69	205	0.34	[0.27; 0.41]
Plunkett 2014	12	50	0.24	[0.13; 0.38]
Quach 2012	27	112	0.24	[0.17; 0.33]
Rajagopalan 2021	13	75	0.17	[0.10; 0.28]
Reddy 2021	26	139	0.19	[0.13; 0.26]
Riaz 2017	10	105	0.10	[0.05; 0.17]
Silva 2020	12	39	0.31	[0.17; 0.48]
Skalitzky 2023	56	286	0.20	[0.15; 0.25]
Vajravelu 2021	801	6163	0.13	[0.12; 0.14]
**Random effects model**		**15425**	**0.14**	**[0.10; 0.20]**

Heterogeneity: $I^2 = 97\%$, $\tau^2 = 0.7583$, $p < 0.01$

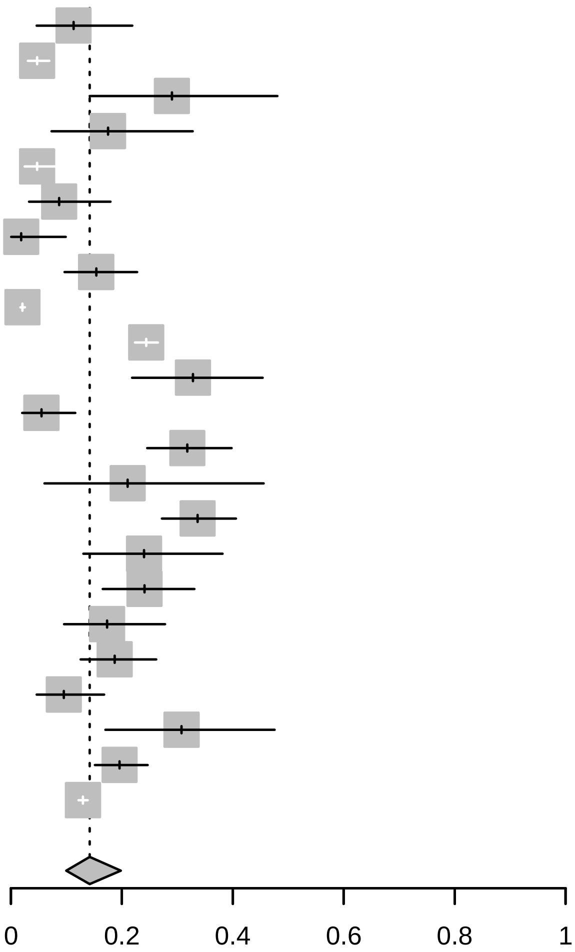

© 2024 Demb J et al. *JAMA Network Open.*

G. Abdominal Distention

Study	Events	Total	Proportion	95%−CI
Chen 2017	5	253	0.02	[0.01; 0.05]
Chou 2011	11	69	0.16	[0.08; 0.27]
Foppa 2021	5	101	0.05	[0.02; 0.11]
Gul 2012	17	50	0.34	[0.21; 0.49]
Law 2017	36	154	0.23	[0.17; 0.31]
Leopa 2023	4	19	0.21	[0.06; 0.46]
Reddy 2021	3	139	0.02	[0.00; 0.06]
Saidi 2018	23	70	0.33	[0.22; 0.45]
Schellerer 2012	39	244	0.16	[0.12; 0.21]
Silva 2020	15	39	0.38	[0.23; 0.55]
Skalitzky 2023	33	286	0.12	[0.08; 0.16]
Zhu 2015	14	83	0.17	[0.10; 0.27]
**Random effects model**		**1507**	**0.14**	**[0.07; 0.24]**

Heterogeneity: $I^2 = 88\%$, $\tau^2 = 1.0558$, $p < 0.01$

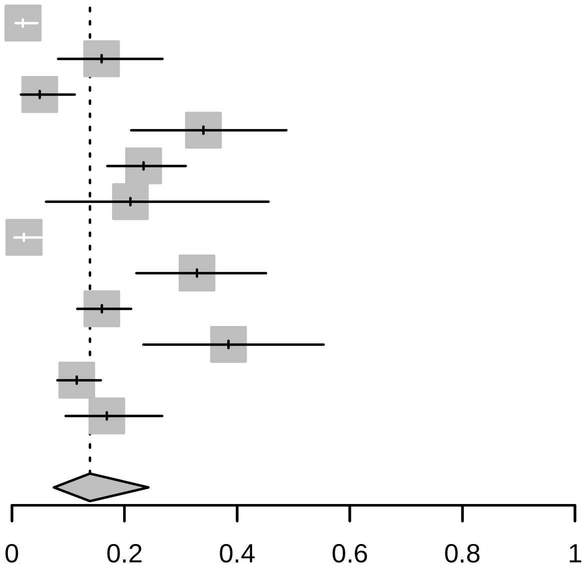

© 2024 Demb J et al. *JAMA Network Open.*

H. Diarrhea

Study	Events	Total	Proportion	95%−CI
Arhi 2019	32	508	0.06	[0.04; 0.09]
Ben−Ishay 2013	5	31	0.16	[0.05; 0.34]
Bouassida 2012	3	40	0.07	[0.02; 0.20]
Chen 2017	15	253	0.06	[0.03; 0.10]
Chou 2011	4	69	0.06	[0.02; 0.14]
DiLeo 2021	5	54	0.09	[0.03; 0.20]
Fritz 2023	140	5075	0.03	[0.02; 0.03]
Glover 2019	390	1680	0.23	[0.21; 0.25]
Jarrar 2022	9	67	0.13	[0.06; 0.24]
Law 2017	45	154	0.29	[0.22; 0.37]
Leopa 2023	5	19	0.26	[0.09; 0.51]
Makmun 2021	46	205	0.22	[0.17; 0.29]
Ozaydin 2019	1	32	0.03	[0.00; 0.16]
Plunkett 2014	10	50	0.20	[0.10; 0.34]
Quach 2012	24	112	0.21	[0.14; 0.30]
Rajagopalan 2021	23	75	0.31	[0.21; 0.42]
Reddy 2021	32	139	0.23	[0.16; 0.31]
Riaz 2017	1	105	0.01	[0.00; 0.05]
Schellerer 2012	20	244	0.08	[0.05; 0.12]
Skalitzky 2023	83	286	0.29	[0.24; 0.35]
Vajravelu 2021	1048	6163	0.17	[0.16; 0.18]
**Random effects model**		**15361**	**0.12**	**[0.08; 0.18]**

Heterogeneity: $I^2 = 97\%$, $\tau^2 = 0.7628$, $p < 0.01$

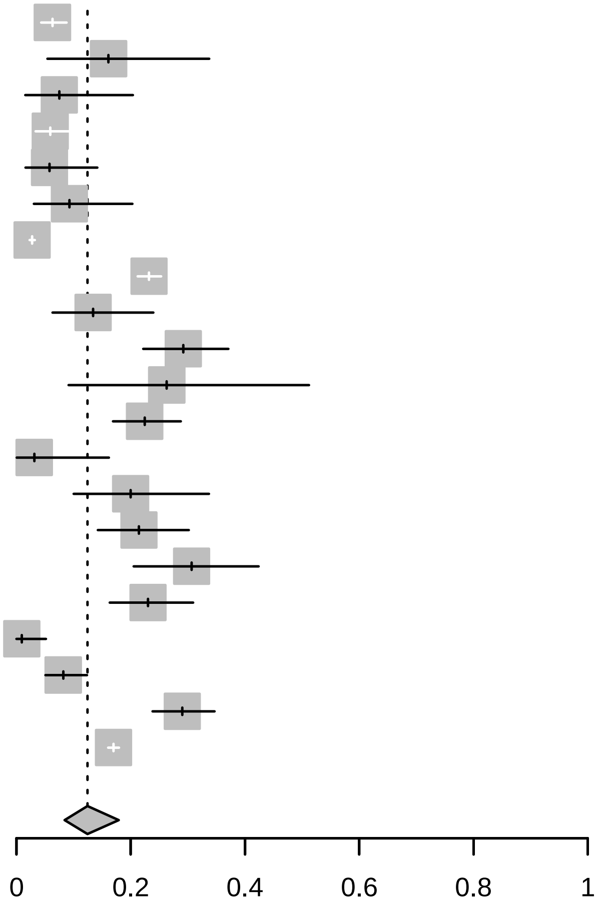

© 2024 Demb J et al. *JAMA Network Open.*

I. Acute Presentation

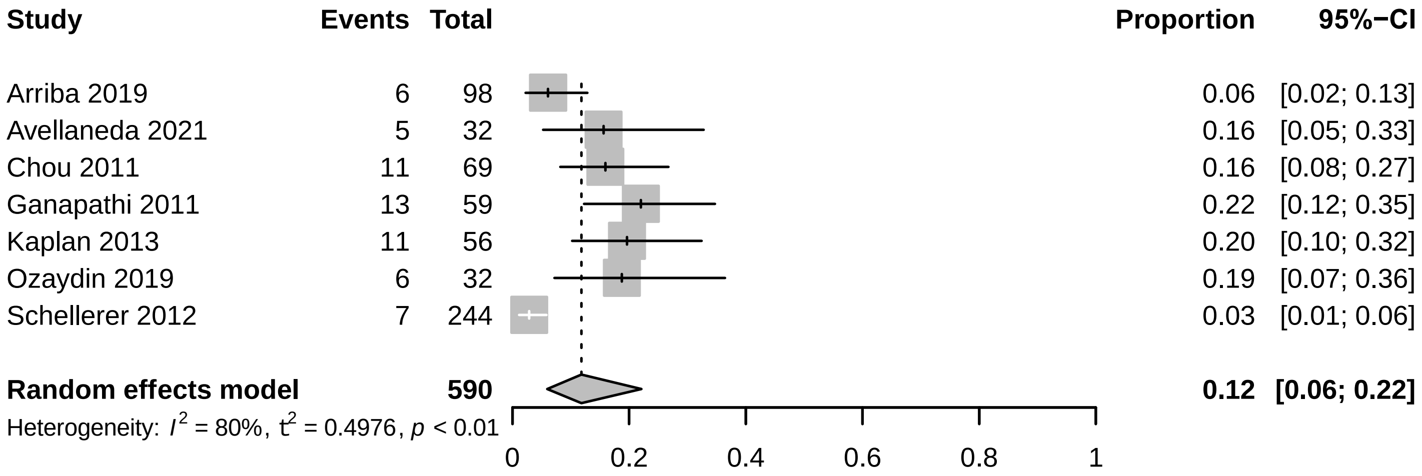

© 2024 Demb J et al. *JAMA Network Open.*

J. Tenesmus

Study	Events	Total	Proportion	95%−CI
Chiu 2023	12	103	0.12	[0.06; 0.19]
Chou 2011	5	69	0.07	[0.02; 0.16]
Gul 2012	26	50	0.52	[0.37; 0.66]
Haleshappa 2017	3	89	0.03	[0.01; 0.10]
Kocian 2017	4	38	0.11	[0.03; 0.25]
Law 2017	19	154	0.12	[0.08; 0.19]
Patel 2016	1	18	0.06	[0.00; 0.27]
Saidi 2018	12	70	0.17	[0.09; 0.28]
Saluja 2014	12	66	0.18	[0.10; 0.30]
Silva 2020	4	39	0.10	[0.03; 0.24]
Wong 2021	10	178	0.06	[0.03; 0.10]
**Random effects model**		**874**	**0.11**	**[0.06; 0.19]**

Heterogeneity: $I^2 = 85\%$, $\tau^2 = 0.6688$, $p < 0.01$

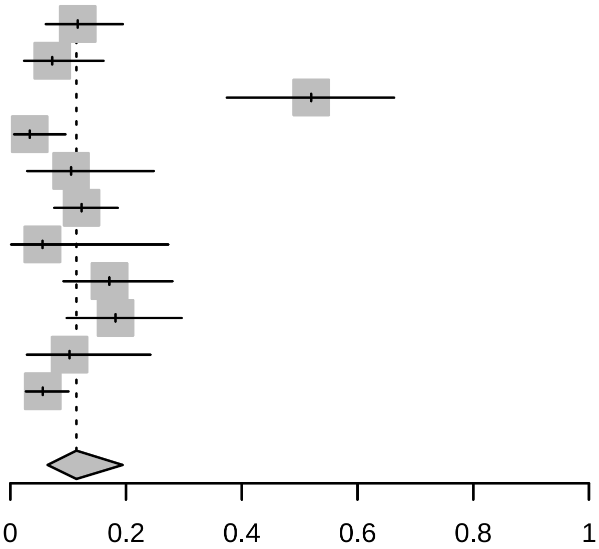

© 2024 Demb J et al. *JAMA Network Open.*

K. Anemia

Study	Events	Total	Proportion	95%−CI
Arhi 2019	20	508	0.04	[0.02; 0.06]
Arriba 2019	5	98	0.05	[0.02; 0.12]
Avellaneda 2021	5	32	0.16	[0.05; 0.33]
Castelo 2023	450	6853	0.07	[0.06; 0.07]
Cercek 2021	25	759	0.03	[0.02; 0.05]
Chiu 2023	15	103	0.15	[0.08; 0.23]
Dharwadkar 2021	31	319	0.10	[0.07; 0.14]
DiLeo 2021	4	54	0.07	[0.02; 0.18]
Fayaz 2018	20	130	0.15	[0.10; 0.23]
Fritz 2023	117	5075	0.02	[0.02; 0.03]
Ganapathi 2011	6	59	0.10	[0.04; 0.21]
Glover 2019	620	1680	0.37	[0.35; 0.39]
Goh 2020	10	65	0.15	[0.08; 0.26]
Gul 2012	36	50	0.72	[0.58; 0.84]
Gunel 2001	5	100	0.05	[0.02; 0.11]
Karsten 2008	7	41	0.17	[0.07; 0.32]
Law 2017	33	154	0.21	[0.15; 0.29]
Leff 2007	7	49	0.14	[0.06; 0.27]
Leopa 2023	12	19	0.63	[0.38; 0.84]
Melendez−Rosado 2022	3	56	0.05	[0.01; 0.15]
Myers 2013	19	180	0.11	[0.06; 0.16]
Nikolic 2023	7	87	0.08	[0.03; 0.16]
Patel 2016	7	18	0.39	[0.17; 0.64]
Quach 2012	3	112	0.03	[0.01; 0.08]
Reddy 2021	24	139	0.17	[0.11; 0.25]
Silva 2020	4	39	0.10	[0.03; 0.24]
Singh 2020	4	22	0.18	[0.05; 0.40]
Strum 2019	6	109	0.06	[0.02; 0.12]
Vajravelu 2021	1541	6163	0.25	[0.24; 0.26]
Vakil 2021	85	637	0.13	[0.11; 0.16]
WanIbrahim 2020	57	893	0.06	[0.05; 0.08]
Wong 2021	11	178	0.06	[0.03; 0.11]
Zhang 2009	4	488	0.01	[0.00; 0.02]
Zhu 2015	38	81	0.47	[0.36; 0.58]
**Random effects model**		**25350**	**0.11**	**[0.08; 0.16]**

Heterogeneity: $I^2 = 98\%$, $\tau^2 = 1.2994$, $p = 0$

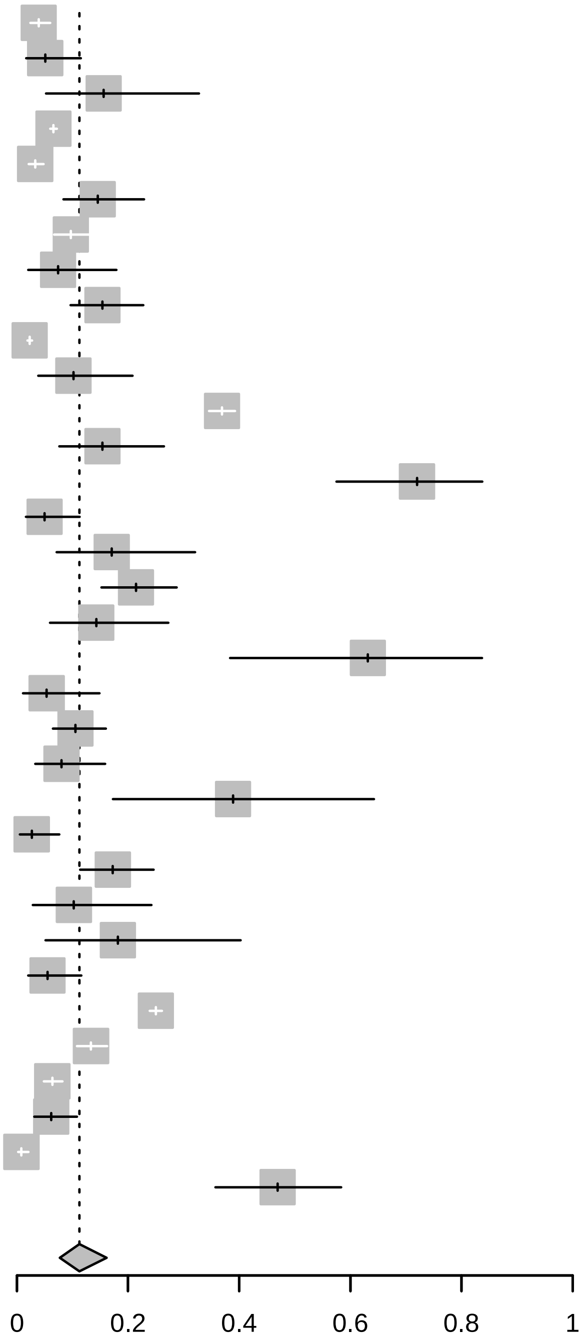

© 2024 Demb J et al. *JAMA Network Open.*

L. Obstruction

Study	Events	Total	Proportion	95%−CI
Al−Barrak 2011	17	62	0.27	[0.17; 0.40]
Bouassida 2012	8	40	0.20	[0.09; 0.36]
Dharwadkar 2021	44	319	0.14	[0.10; 0.18]
El−Hennawy 2003	4	26	0.15	[0.04; 0.35]
Fayaz 2018	18	130	0.14	[0.08; 0.21]
Goh 2020	20	30	0.67	[0.47; 0.83]
Haleshappa 2017	13	89	0.15	[0.08; 0.24]
Jarrar 2022	14	67	0.21	[0.12; 0.33]
Karsten 2008	4	41	0.10	[0.03; 0.23]
Lin 2005	1	45	0.02	[0.00; 0.12]
Nagai 2016	5	70	0.07	[0.02; 0.16]
Park 2022	105	3856	0.03	[0.02; 0.03]
Riaz 2017	18	105	0.17	[0.10; 0.26]
Saidi 2018	10	70	0.14	[0.07; 0.25]
Saluja 2014	17	66	0.26	[0.16; 0.38]
Silva 2019	136	781	0.17	[0.15; 0.20]
Singh 2020	4	22	0.18	[0.05; 0.40]
WanIbrahim 2020	66	893	0.07	[0.06; 0.09]
Wong 2021	32	178	0.18	[0.13; 0.24]
Zahir 2014	29	131	0.22	[0.15; 0.30]
Cercek 2021	4	759	0.01	[0.00; 0.01]
Melendez−Rosado 2022	7	56	0.12	[0.05; 0.24]
Ozaydin 2019	1	32	0.03	[0.00; 0.16]
Ruiz 2016	33	196	0.17	[0.12; 0.23]
Skalitzky 2023	16	286	0.06	[0.03; 0.09]
Trivedi 2022	9	148	0.06	[0.03; 0.11]
Vakil 2021	17	637	0.03	[0.02; 0.04]
**Random effects model**		**9135**	**0.11**	**[0.07; 0.16]**

Heterogeneity: $I^2 = 95\%$, $\tau^2 = 1.0821$, $p < 0.01$

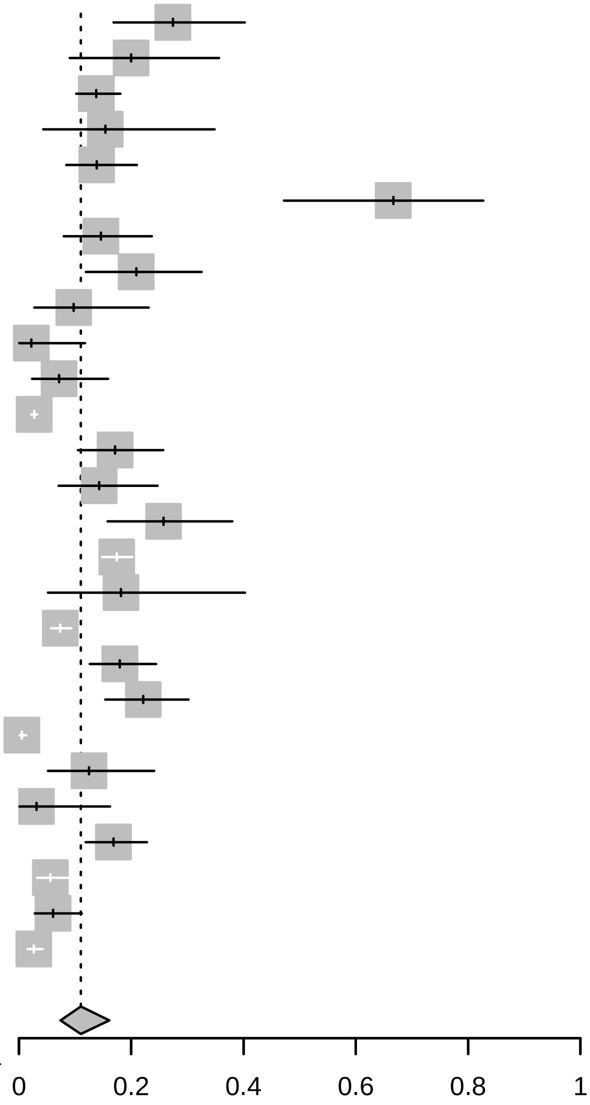

© 2024 Demb J et al. *JAMA Network Open.*

M. Perforation

Study	Events	Total	Proportion	95%−CI
Kocian 2017	1	38	0.03	[0.00; 0.14]
Myers 2013	5	180	0.03	[0.01; 0.06]
Ruiz 2016	7	196	0.04	[0.01; 0.07]
Trivedi 2022	3	148	0.02	[0.00; 0.06]
Al−Barrak 2011	17	62	0.27	[0.17; 0.40]
Goh 2020	20	30	0.67	[0.47; 0.83]
Law 2017	61	154	0.40	[0.32; 0.48]
Lin 2005	1	45	0.02	[0.00; 0.12]
Nagai 2016	5	70	0.07	[0.02; 0.16]
Singh 2020	4	22	0.18	[0.05; 0.40]
**Random effects model**		**945**	**0.09**	**[0.03; 0.25]**

Heterogeneity: $I^2 = 94\%$, $\tau^2 = 2.3948$, $p < 0.01$

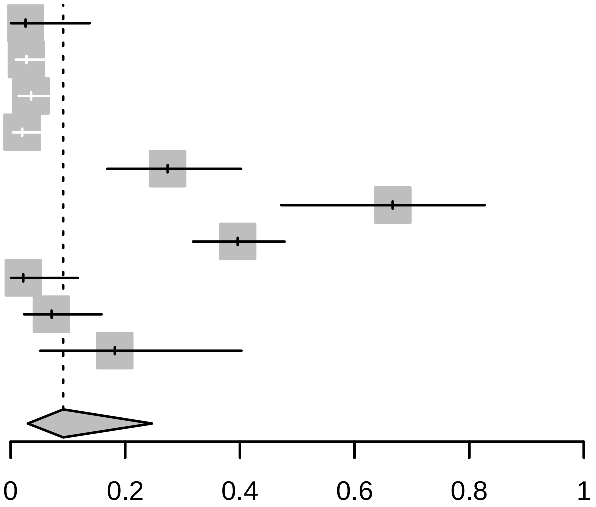

© 2024 Demb J et al. *JAMA Network Open.*

N. Fatigue

Study	Events	Total	Proportion	95%−CI
Arhi 2019	10	508	0.02	[0.01; 0.04]
Bouassida 2012	11	40	0.28	[0.15; 0.44]
Cercek 2021	28	759	0.04	[0.02; 0.05]
Chen 2017	8	253	0.03	[0.01; 0.06]
DiLeo 2021	1	54	0.02	[0.00; 0.10]
Fritz 2023	324	5075	0.06	[0.06; 0.07]
Glover 2019	280	1680	0.17	[0.15; 0.19]
Goh 2020	9	65	0.14	[0.07; 0.25]
Park 2022	147	3856	0.04	[0.03; 0.04]
Rajagopalan 2021	9	75	0.12	[0.06; 0.22]
Saluja 2014	13	66	0.20	[0.11; 0.31]
Schellerer 2012	39	244	0.16	[0.12; 0.21]
Silva 2020	3	39	0.08	[0.02; 0.21]
Skalitzky 2023	45	286	0.16	[0.12; 0.20]
Zhu 2015	12	83	0.14	[0.08; 0.24]
**Random effects model**		**13083**	**0.08**	**[0.05; 0.13]**

Heterogeneity: $I^2 = 96\%$, $\tau^2 = 0.6984$, $p < 0.01$

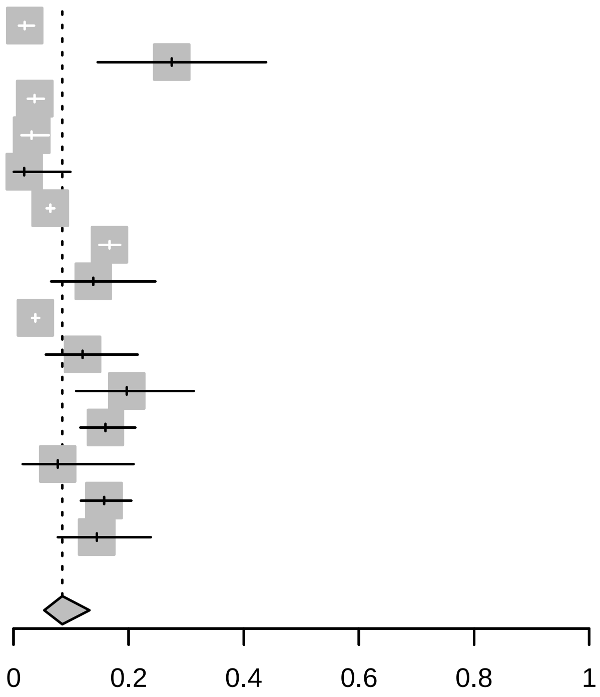

© 2024 Demb J et al. *JAMA Network Open.*

O. Nausea/Vomiting

Study	Events	Total	Proportion	95%−CI
Ben−Ishay 2013	6	31	0.19	[0.07; 0.37]
Chen 2017	2	253	0.01	[0.00; 0.03]
Chou 2011	2	69	0.03	[0.00; 0.10]
Fritz 2023	143	5075	0.03	[0.02; 0.03]
Glover 2019	560	1680	0.33	[0.31; 0.36]
Kaplan 2013	7	56	0.12	[0.05; 0.24]
Kaplan 2019	10	141	0.07	[0.03; 0.13]
Limaiem 2018	1	32	0.03	[0.00; 0.16]
Plunkett 2014	13	50	0.26	[0.15; 0.40]
Raman 2014	3	72	0.04	[0.01; 0.12]
Reddy 2021	15	139	0.11	[0.06; 0.17]
Silva 2020	9	39	0.23	[0.11; 0.39]
**Random effects model**		**7637**	**0.08**	**[0.04; 0.16]**

Heterogeneity: $I^2 = 99\%$, $\tau^2 = 1.2948$, $p < 0.01$

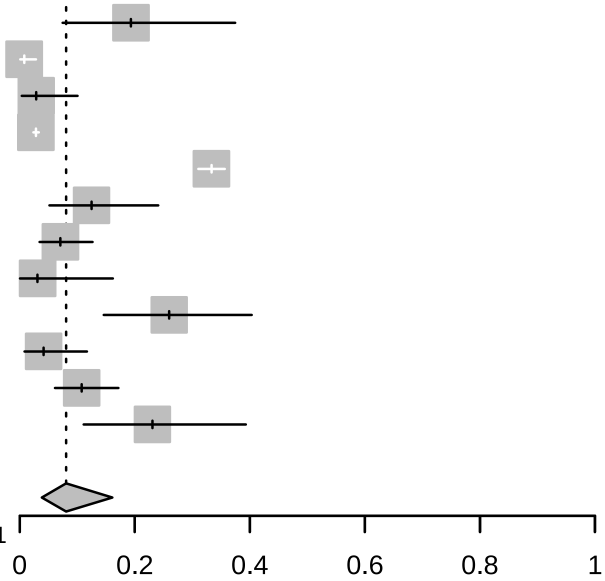

© 2024 Demb J et al. *JAMA Network Open.*

P. Abdominal Mass

Study	Events	Total	Proportion	95%−CI
Arhi 2019	3	508	0.01	[0.00; 0.02]
Chiu 2023	3	103	0.03	[0.01; 0.08]
deSilva 2000	4	60	0.07	[0.02; 0.16]
Gul 2012	17	50	0.34	[0.21; 0.49]
Haroon 2013	3	23	0.13	[0.03; 0.34]
Limaiem 2018	4	32	0.12	[0.04; 0.29]
Lin 2005	3	45	0.07	[0.01; 0.18]
Poudyal 2017	4	30	0.13	[0.04; 0.31]
Saluja 2014	13	66	0.20	[0.11; 0.31]
Silva 2020	2	39	0.05	[0.01; 0.17]
Vakil 2021	31	637	0.05	[0.03; 0.07]
Zahir 2014	16	131	0.12	[0.07; 0.19]
Zhu 2015	7	83	0.08	[0.03; 0.17]
**Random effects model**		**1807**	**0.08**	**[0.04; 0.14]**

Heterogeneity: $I^2 = 86\%$, $\tau^2 = 0.9595$, $p < 0.01$

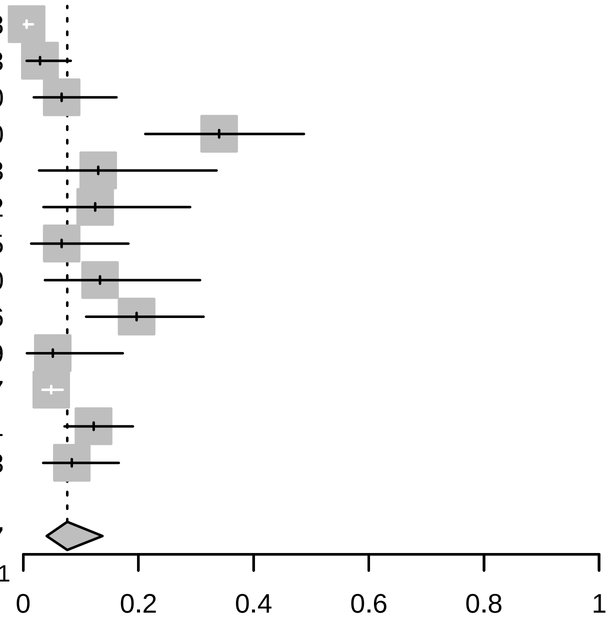

© 2024 Demb J et al. *JAMA Network Open.*

Q. Rectal Pain

Study	Events	Total	Proportion	95%−CI
Cercek 2021	136	759	0.18	[0.15; 0.21]
Ganapathi 2011	1	59	0.02	[0.00; 0.09]
Lapumnuaypol 2018	6	109	0.06	[0.02; 0.12]
Melendez−Rosado 2022	6	56	0.11	[0.04; 0.22]
Scott 2016	2	56	0.04	[0.00; 0.12]
Skalitzky 2023	21	286	0.07	[0.05; 0.11]
Syed 2019	170	5710	0.03	[0.03; 0.03]
Vakil 2021	45	637	0.07	[0.05; 0.09]
Myers 2013	7	180	0.04	[0.02; 0.08]
Park 2022	97	3856	0.03	[0.02; 0.03]
Reddy 2021	3	139	0.02	[0.00; 0.06]
Silva 2020	1	39	0.03	[0.00; 0.13]
**Random effects model**		**11886**	**0.05**	**[0.03; 0.08]**

Heterogeneity: $I^2 = 97\%$, $\tau^2 = 0.4515$, $p < 0.01$

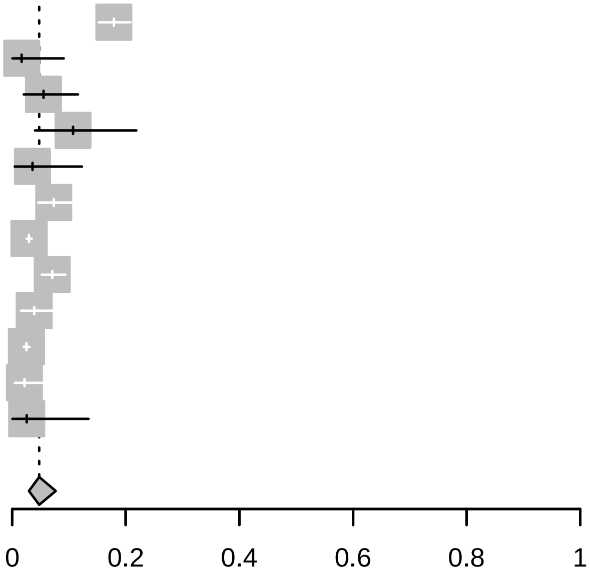

© 2024 Demb J et al. *JAMA Network Open.*

eFigure 2. Pooled proportions of presenting signs and symptoms for EOCRC by geography

A. In the United States

Sign/Symptom	Number of Studies	Proportion	Weighted Proportion (95% CI)
Hematochezia	20	8391/28452	0.40 [0.31; 0.50]
Abdominal Pain	17	9762/25475	0.35 [0.28; 0.42]
Altered Bowel Habits	16	4078/19229	0.16 [0.10; 0.25]
Diarrhea	6	1708/13596	0.13 [0.07; 0.25]
Tenesmus	1	12/103	0.12 [0.07; 0.19]
Anemia	12	2493/15261	0.11 [0.06; 0.17]
Weight Loss	13	1517/19328	0.10 [0.07; 0.14]
Constipation	7	1417/13705	0.10 [0.05; 0.18]
Fatigue	6	832/11909	0.07 [0.04; 0.12]
Nausea/Vomiting	4	720/7147	0.06 [0.01; 0.23]
Loss of Appetite	3	90/2075	0.06 [0.03; 0.11]
Rectal Pain	10	493/11788	0.05 [0.03; 0.08]
Abdominal Mass	2	34/740	0.05 [0.03; 0.06]
Obstruction	7	197/5954	0.04 [0.02; 0.09]
Abdominal Distension	3	41/678	0.04 [0.01; 0.11]
Perforation	1	5/180	0.03 [0.01; 0.06]

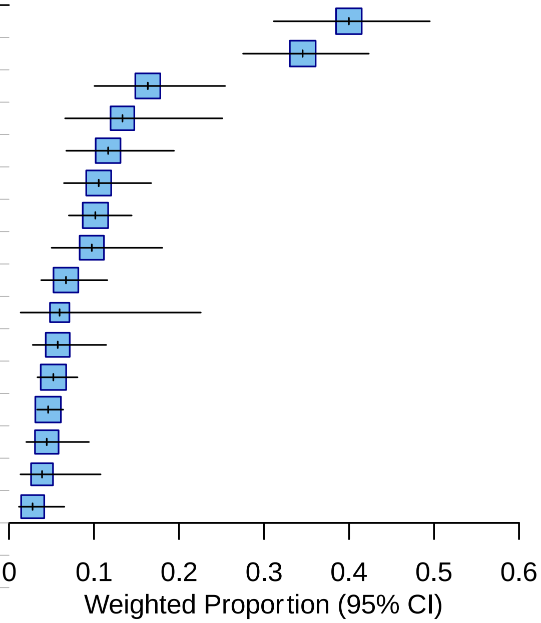

B. Outside the United States

Sign/Symptom	Number of Studies	Proportion	Weighted Proportion (95% CI)
Hematochezia	56	2928/6979	0.47 [0.41; 0.53]
Abdominal Pain	56	2765/6972	0.42 [0.35; 0.49]
Altered Bowel Habits	47	1659/5431	0.32 [0.26; 0.38]
Loss of Appetite	6	144/1138	0.24 [0.08; 0.55]
Abdominal Distension	9	164/829	0.20 [0.14; 0.29]
Weight Loss	40	1162/5747	0.19 [0.13; 0.27]
Constipation	16	292/1720	0.17 [0.12; 0.23]
Obstruction	20	455/3181	0.15 [0.11; 0.20]
Diarrhea	15	233/1765	0.12 [0.08; 0.18]
Acute Presentation	7	59/590	0.12 [0.07; 0.20]
Anemia	22	748/10089	0.12 [0.07; 0.19]
Tenesmus	10	96/771	0.11 [0.07; 0.19]
Perforation	9	119/765	0.11 [0.04; 0.26]
Fatigue	9	107/1174	0.10 [0.06; 0.17]
Nausea/Vomiting	8	51/490	0.10 [0.05; 0.17]
Abdominal Mass	11	76/1067	0.09 [0.05; 0.16]
Rectal Pain	2	2/98	0.02 [0.01; 0.08]

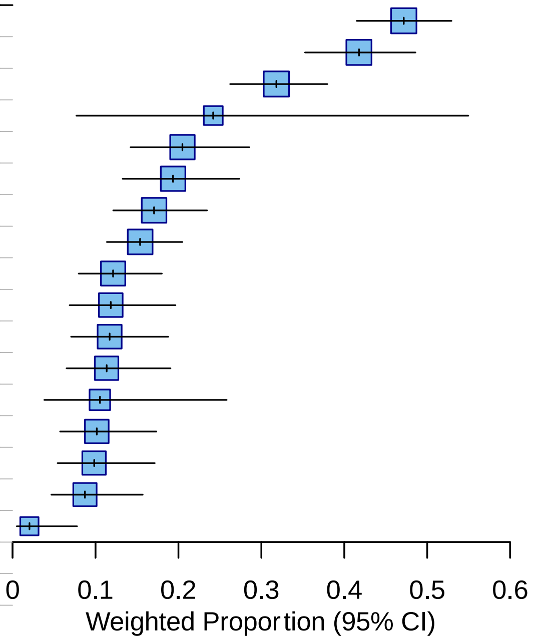

© 2024 Demb J et al. *JAMA Network Open.*

eFigure 3. Pooled proportions of presenting signs and symptoms for EOCRC; stratified analysis by age group

A. Studies including adults age ≤50.

Sign/Symptom	Number of Studies	Proportion	Weighted Proportion (95% CI)
Hematochezia	41	9680/30902	0.41 [0.35; 0.48]
Abdominal Pain	39	10406/27981	0.37 [0.30; 0.44]
Altered Bowel Habits	33	4230/20726	0.23 [0.16; 0.30]
Weight Loss	25	1965/20875	0.15 [0.10; 0.22]
Abdominal Distension	5	128/976	0.14 [0.06; 0.29]
Perforation	6	98/604	0.13 [0.04; 0.37]
Constipation	15	1224/13291	0.13 [0.08; 0.20]
Diarrhea	13	1473/13257	0.11 [0.07; 0.17]
Obstruction	15	508/8281	0.09 [0.05; 0.17]
Tenesmus	5	46/492	0.09 [0.07; 0.13]
Anemia	23	2477/22601	0.09 [0.06; 0.12]
Loss of Appetite	4	96/1327	0.08 [0.05; 0.11]
Fatigue	10	614/11139	0.06 [0.04; 0.09]
Nausea/Vomiting	5	163/5470	0.05 [0.02; 0.16]
Rectal Pain	9	485/11632	0.05 [0.03; 0.08]
Abdominal Mass	4	39/1287	0.02 [0.01; 0.06]

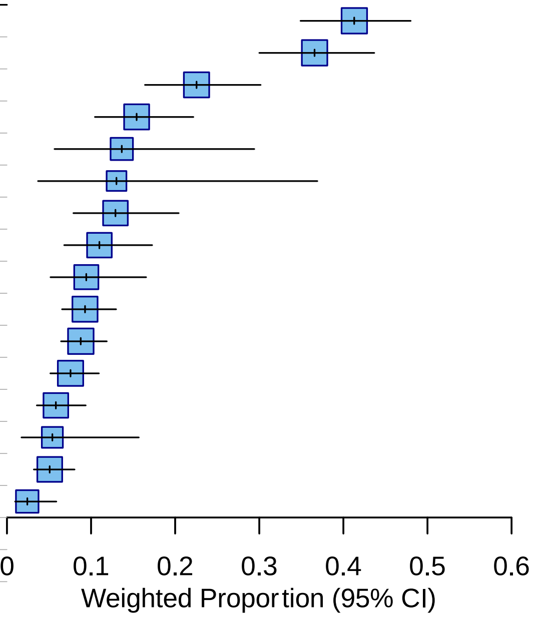

B. Studies including adults ages ≤40

Sign/Symptom	Number of Studies	Proportion	Weighted Proportion (95% CI)
Hematochezia	24	919/3127	0.50 [0.40; 0.60]
Abdominal Pain	23	1491/3064	0.41 [0.31; 0.52]
Altered Bowel Habits	23	1221/2981	0.32 [0.24; 0.41]
Loss of Appetite	5	138/1886	0.26 [0.06; 0.65]
Anemia	9	722/2180	0.22 [0.11; 0.39]
Constipation	5	453/1947	0.19 [0.13; 0.25]
Fatigue	3	304/1786	0.17 [0.15; 0.19]
Diarrhea	5	434/1947	0.16 [0.09; 0.26]
Weight Loss	20	436/2954	0.15 [0.08; 0.27]
Obstruction	9	97/629	0.15 [0.11; 0.19]
Abdominal Mass	7	48/306	0.14 [0.09; 0.22]
Tenesmus	6	62/382	0.14 [0.06; 0.28]
Abdominal Distension	6	63/448	0.14 [0.06; 0.29]
Nausea/Vomiting	4	578/1920	0.09 [0.03; 0.26]
Rectal Pain	3	10/254	0.04 [0.01; 0.10]
Perforation	3	9/279	0.03 [0.02; 0.06]

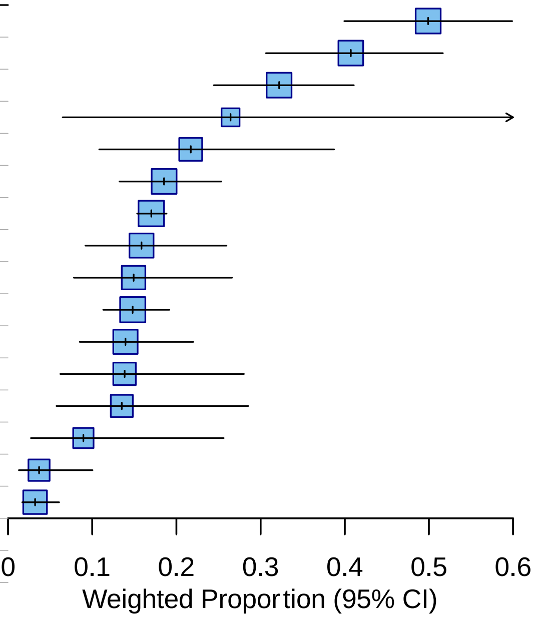

© 2024 Demb J et al. *JAMA Network Open.*

eFigure 4. Pooled proportions of presenting signs and symptoms for EOCRC, stratified analysis by risk of bias.

A. Studies with low risk of bias.

Sign/Symptom	Number of Studies	Proportion	Weighted Proportion (95% CI)
Hematochezia	63	10879/34543	0.44 [0.39; 0.50]
Abdominal Pain	61	12194/31585	0.40 [0.34; 0.46]
Altered Bowel Habits	52	5543/23903	0.27 [0.22; 0.33]
Weight Loss	45	2509/24530	0.16 [0.11; 0.21]
Diarrhea	16	1904/15004	0.14 [0.10; 0.20]
Constipation	18	1650/15068	0.14 [0.09; 0.20]
Abdominal Distension	10	177/1388	0.12 [0.07; 0.22]
Anemia	29	3186/24931	0.11 [0.08; 0.16]
Acute Presentation	4	29/364	0.11 [0.05; 0.23]
Loss of Appetite	6	166/3037	0.10 [0.04; 0.25]
Obstruction	23	605/8898	0.10 [0.06; 0.15]
Tenesmus	8	65/689	0.09 [0.06; 0.13]
Perforation	10	124/945	0.09 [0.04; 0.22]
Nausea/Vomiting	9	760/7465	0.09 [0.04; 0.18]
Fatigue	13	915/12977	0.07 [0.05; 0.11]
Abdominal Mass	10	76/1631	0.06 [0.03; 0.10]
Rectal Pain	11	494/11827	0.05 [0.03; 0.08]

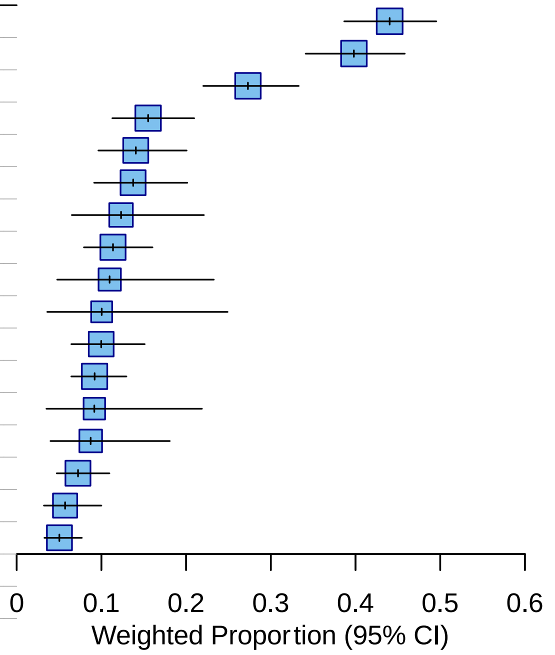

B. Studies with moderate risk of bias.

Sign/Symptom	Number of Studies	Proportion	Weighted Proportion (95% CI)
Hematochezia	9	265/595	0.43 [0.34; 0.53]
Abdominal Pain	9	203/595	0.36 [0.26; 0.48]
Obstruction	2	25/106	0.24 [0.16; 0.33]
Altered Bowel Habits	9	140/595	0.23 [0.16; 0.33]
Fatigue	2	24/106	0.23 [0.16; 0.32]
Loss of Appetite	2	33/126	0.18 [0.03; 0.61]
Constipation	3	22/140	0.16 [0.09; 0.28]
Abdominal Distension	1	11/69	0.16 [0.09; 0.27]
Weight Loss	6	82/383	0.15 [0.05; 0.36]
Acute Presentation	3	30/226	0.13 [0.07; 0.23]
Abdominal Mass	2	17/126	0.12 [0.06; 0.25]
Tenesmus	2	17/135	0.12 [0.06; 0.22]
Diarrhea	3	12/140	0.09 [0.05; 0.14]
Nausea/Vomiting	3	11/172	0.06 [0.02; 0.17]
Anemia	3	16/257	0.06 [0.04; 0.10]
Rectal Pain	1	1/59	0.02 [0.00; 0.11]

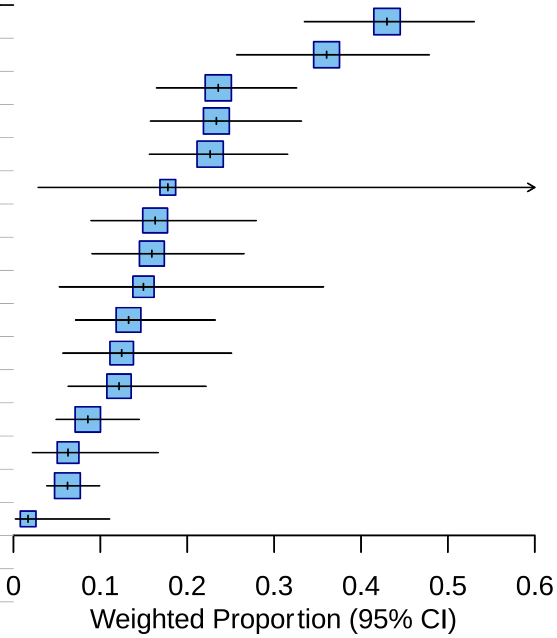

© 2024 Demb J et al. *JAMA Network Open.*

C. Studies with high risk of bias

Sign/Symptom	Number of Studies	Proportion	Weighted Proportion (95% CI)
Loss of Appetite	1	35/50	0.70 [0.56; 0.81]
Hematochezia	4	175/293	0.70 [0.46; 0.86]
Weight Loss	2	88/162	0.65 [0.31; 0.89]
Abdominal Pain	3	130/267	0.54 [0.20; 0.85]
Tenesmus	1	26/50	0.52 [0.38; 0.65]
Altered Bowel Habits	2	54/162	0.45 [0.04; 0.93]
Abdominal Mass	1	17/50	0.34 [0.22; 0.48]
Abdominal Distension	1	17/50	0.34 [0.22; 0.48]
Anemia	2	39/162	0.21 [0.01; 0.87]
Obstruction	2	22/131	0.17 [0.11; 0.24]
Constipation	2	37/217	0.16 [0.08; 0.29]
Diarrhea	2	25/217	0.05 [0.00; 0.40]

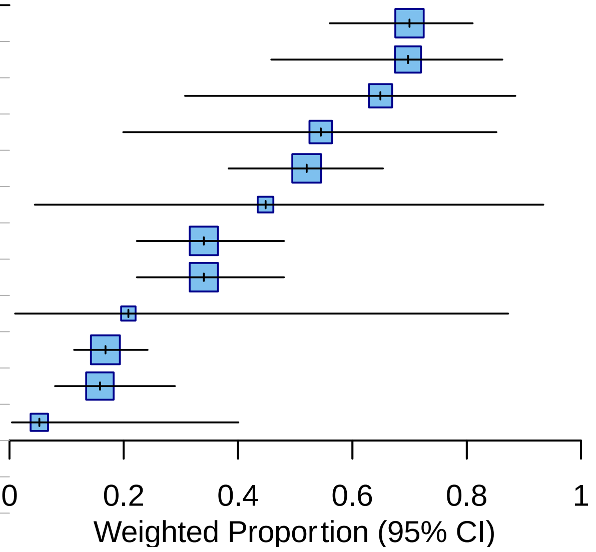

© 2024 Demb J et al. *JAMA Network Open.*

eFigure 5. Pooled proportions of presenting signs and symptoms for EOCRC, stratified analysis by data source.

A. Claims or Medical Records

Sign/Symptom	Number of Studies	Proportion	Weighted Proportion (95% CI)
Hematochezia	63	10879/34543	0.44 [0.39; 0.50]
Abdominal Pain	61	12194/31585	0.40 [0.34; 0.46]
Altered Bowel Habits	52	5543/23903	0.27 [0.22; 0.33]
Weight Loss	45	2509/24530	0.16 [0.11; 0.21]
Diarrhea	16	1904/15004	0.14 [0.10; 0.20]
Constipation	18	1650/15068	0.14 [0.09; 0.20]
Abdominal Distension	10	177/1388	0.12 [0.07; 0.22]
Anemia	29	3186/24931	0.11 [0.08; 0.16]
Acute Presentation	4	29/364	0.11 [0.05; 0.23]
Loss of Appetite	6	166/3037	0.10 [0.04; 0.25]
Obstruction	23	605/8898	0.10 [0.06; 0.15]
Tenesmus	8	65/689	0.09 [0.06; 0.13]
Perforation	10	124/945	0.09 [0.04; 0.22]
Nausea/Vomiting	9	760/7465	0.09 [0.04; 0.18]
Fatigue	13	915/12977	0.07 [0.05; 0.11]
Abdominal Mass	10	76/1631	0.06 [0.03; 0.10]
Rectal Pain	11	494/11827	0.05 [0.03; 0.08]

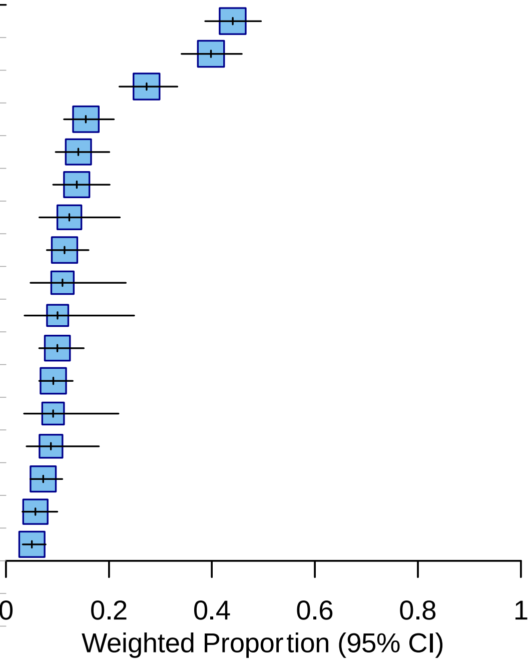

B. Patient-Reported

Sign/Symptom	Number of Studies	Proportion	Weighted Proportion (95% CI)
Hematochezia	6	201/425	0.45 [0.35; 0.56]
Abdominal Pain	6	109/425	0.28 [0.18; 0.41]
Altered Bowel Habits	5	74/320	0.24 [0.16; 0.35]
Obstruction	1	18/105	0.17 [0.11; 0.26]
Constipation	2	19/136	0.16 [0.07; 0.33]
Acute Presentation	2	19/157	0.12 [0.05; 0.28]
Weight Loss	4	34/248	0.10 [0.03; 0.27]
Nausea/Vomiting	2	9/103	0.09 [0.03; 0.25]
Anemia	2	11/157	0.07 [0.04; 0.12]
Abdominal Mass	1	4/60	0.07 [0.03; 0.16]
Loss of Appetite	1	3/60	0.05 [0.02; 0.14]
Diarrhea	2	6/136	0.04 [0.00; 0.28]
Rectal Pain	1	1/59	0.02 [0.00; 0.11]

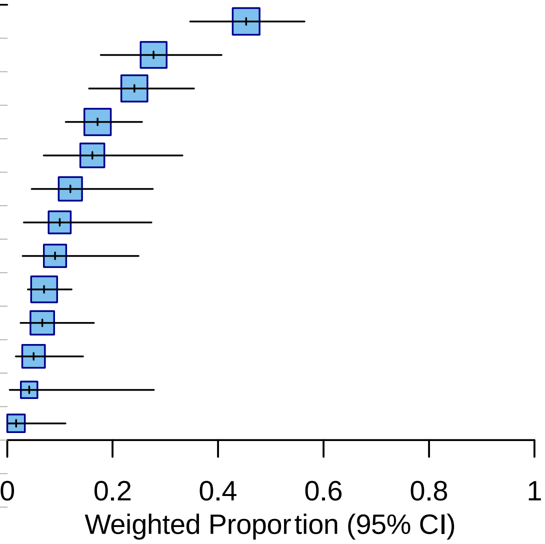

© 2024 Demb J et al. *JAMA Network Open.*

C. Not well defined

Sign/Symptom	Number of Studies	Proportion	Weighted Proportion (95% CI)
Loss of Appetite	2	65/116	0.58 [0.40; 0.74]
Hematochezia	7	239/463	0.57 [0.37; 0.75]
Abdominal Pain	6	224/437	0.54 [0.36; 0.71]
Weight Loss	4	136/297	0.48 [0.20; 0.77]
Altered Bowel Habits	6	120/437	0.28 [0.11; 0.56]
Abdominal Mass	2	30/116	0.26 [0.17; 0.37]
Abdominal Distension	2	28/119	0.24 [0.13; 0.38]
Fatigue	2	24/106	0.23 [0.16; 0.32]
Obstruction	3	29/132	0.22 [0.16; 0.30]
Tenesmus	3	43/185	0.21 [0.07; 0.48]
Constipation	3	40/221	0.16 [0.10; 0.26]
Acute Presentation	1	11/69	0.16 [0.09; 0.27]
Anemia	3	44/262	0.13 [0.01; 0.62]
Diarrhea	3	31/221	0.11 [0.05; 0.22]
Nausea/Vomiting	1	2/69	0.03 [0.01; 0.11]

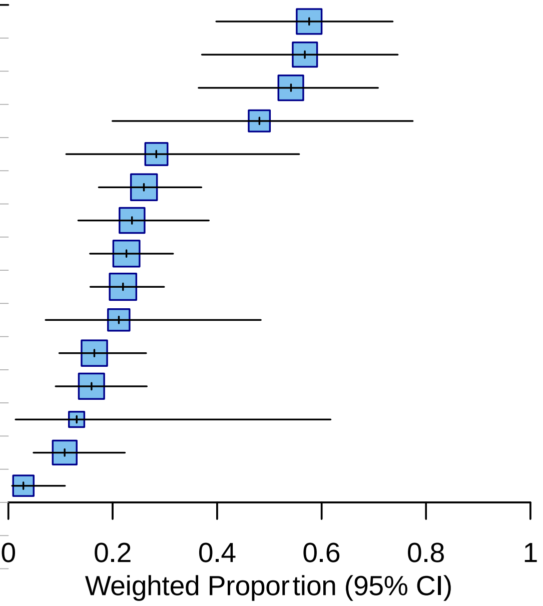

© 2024 Demb J et al. *JAMA Network Open.*

eFigure 6. Histograms of mean and median diagnosis stratified by data source.

A. Stratified histograms by data source

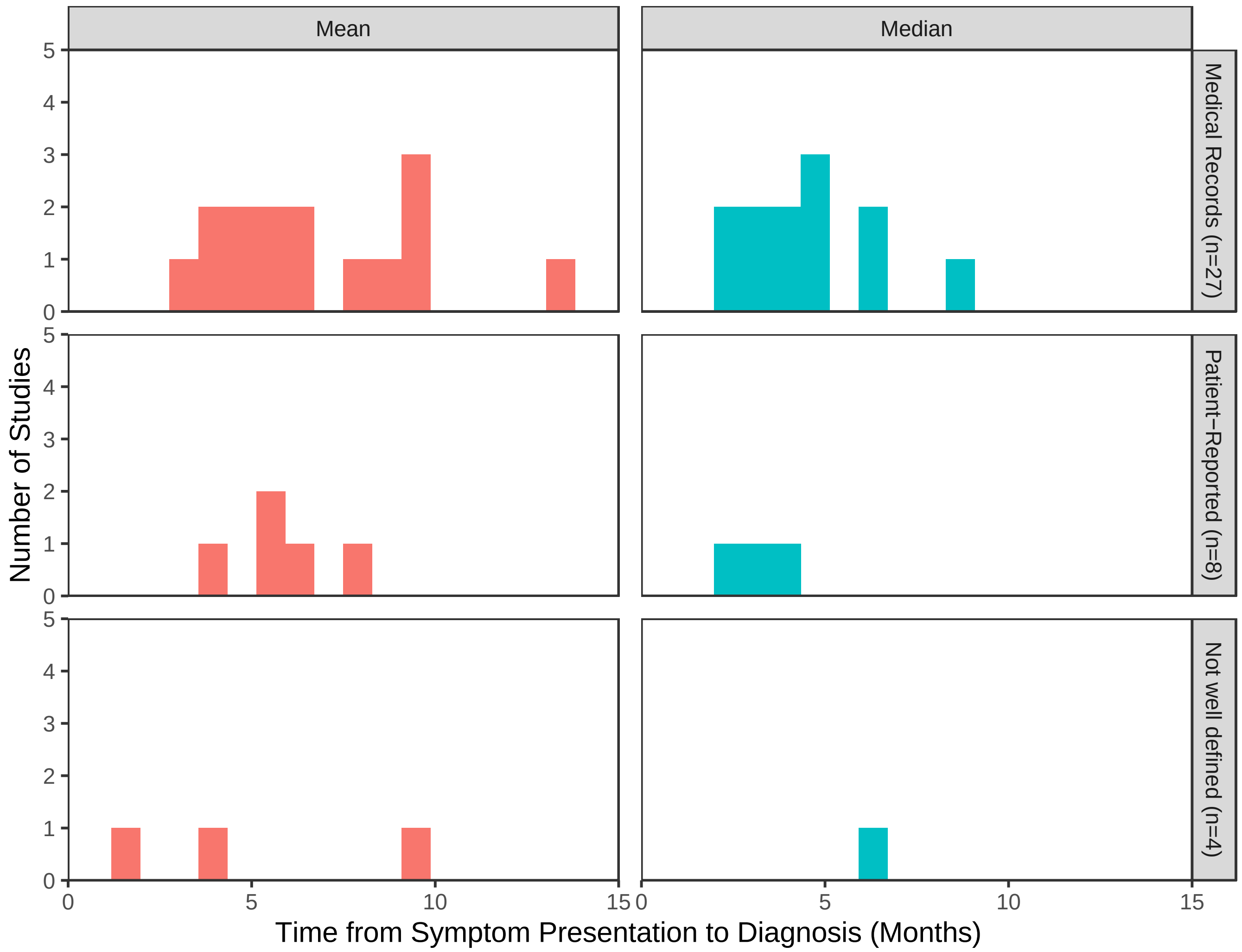

Medical records (Mean: 6.8 months, Range: 3.0-13.7 months; Median: 4.1 months, Range: 2.0-8.7 months)
Patient-reported (Mean: 5.9 months, Range: 4.2-8.1 months; Median: 3 months, Range: 2.0-4.3 months)
Not well defined (Mean: 5.2 months, Range: 1.8-9.8 months; Median: 6 months)

© 2024 Demb J et al. *JAMA Network Open.*

B. Overall histogram excluding "not well defined" data sources.

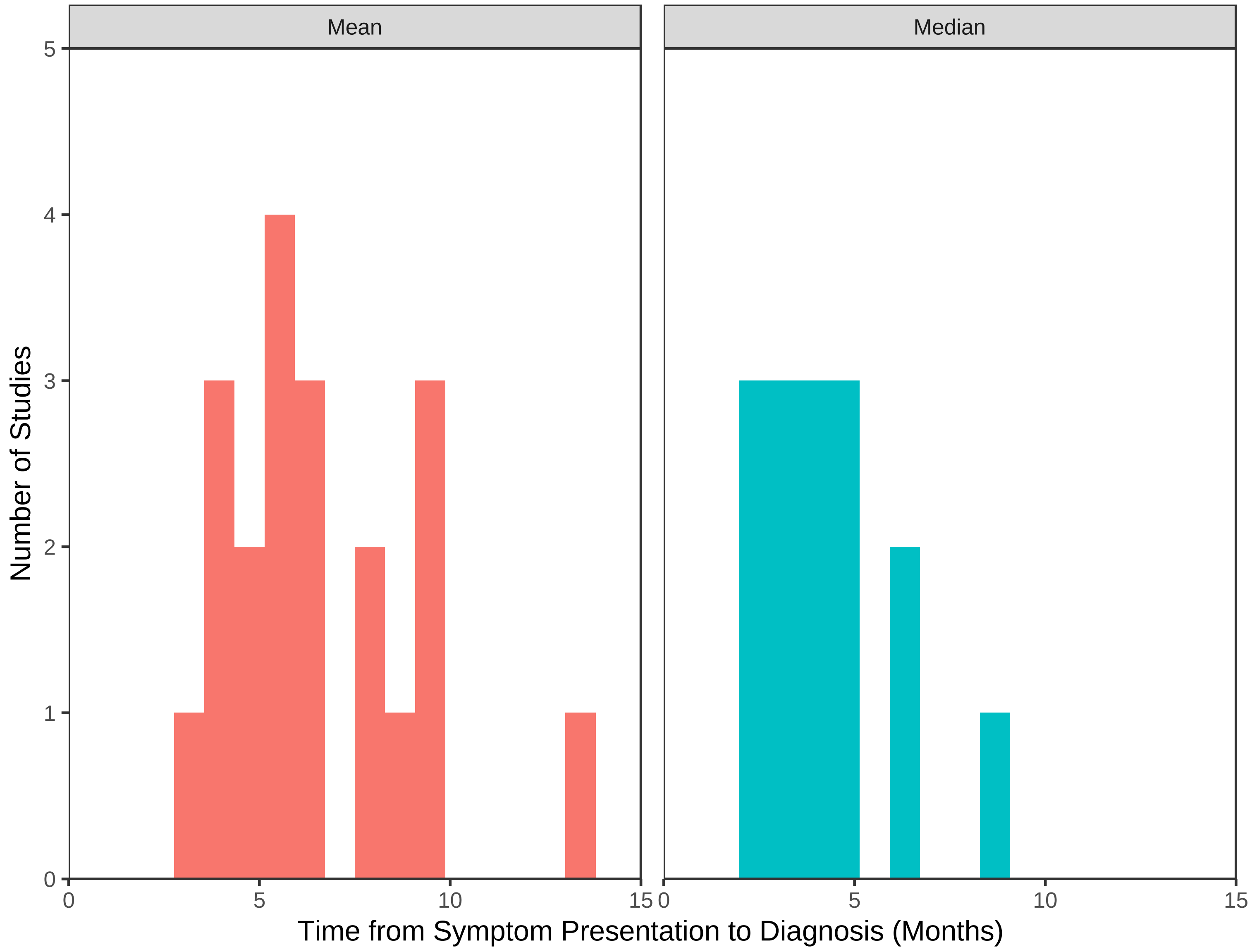

© 2024 Demb J et al. *JAMA Network Open.*
