# Supplementary material for: Red Flag Signs and Symptoms for Patients With Early-Onset Colorectal Cancer: A Systematic Review and Meta-Analysis
Source: JAMA Netw Open. 2024 May 24;7(5):e2413157. doi: 10.1001/jamanetworkopen.2024.13157 (PMC11127127; doi:10.1001/jamanetworkopen.2024.13157)
Supplement: Supplement 2. — Data Sharing Statement [file jamanetwopen-e2413157-s002.pdf]

Data Sharing Statement

Demb. Red Flag Signs and Symptoms for Patients With Early-Onset Colorectal Cancer. *JAMA Netw Open*. Published May 24, 2024. doi:10.1001/jamanetworkopen.2024.13157

Data
Data available: No

Additional Information
Explanation for why data not available: Data for systematic review and meta-analysis were abstracted from peer-reviewed studies. We have provided the list of all studies included in our review and their reference, as well as the relevant data we extracted from each study in our tables, figures and supplementary materials.
